# Artificial pancreas treatment for outpatients with type 1 diabetes: systematic review and meta-analysis

**DOI:** 10.1136/bmj.k1310

**Published:** 2018-04-18

**Authors:** Eleni Bekiari, Konstantinos Kitsios, Hood Thabit, Martin Tauschmann, Eleni Athanasiadou, Thomas Karagiannis, Anna-Bettina Haidich, Roman Hovorka, Apostolos Tsapas

**Affiliations:** 1 Clinical Research and Evidence Based Medicine Unit, Aristotle University of Thessaloniki, 54642 Thessaloniki, Greece; 2 Diabetes Centre, Second Medical Department, Aristotle University of Thessaloniki, Thessaloniki, Greece; 3 Wellcome Trust-Medical Research Council Institute of Metabolic Science, University of Cambridge, Cambridge, UK; 4 Department of Hygiene and Epidemiology, Medical School, Aristotle University of Thessaloniki, Thessaloniki, Greece; 5 Harris Manchester College, University of Oxford, Oxford, UK

## Abstract

**Objective:**

To evaluate the efficacy and safety of artificial pancreas treatment in non-pregnant outpatients with type 1 diabetes.

**Design:**

Systematic review and meta-analysis of randomised controlled trials.

**Data sources:**

Medline, Embase, Cochrane Library, and grey literature up to 2 February 2018.

**Eligibility criteria for selecting studies:**

Randomised controlled trials in non-pregnant outpatients with type 1 diabetes that compared the use of any artificial pancreas system with any type of insulin based treatment. Primary outcome was proportion (%) of time that sensor glucose level was within the near normoglycaemic range (3.9-10 mmol/L). Secondary outcomes included proportion (%) of time that sensor glucose level was above 10 mmol/L or below 3.9 mmol/L, low blood glucose index overnight, mean sensor glucose level, total daily insulin needs, and glycated haemoglobin. The Cochrane Collaboration risk of bias tool was used to assess study quality.

**Results:**

40 studies (1027 participants with data for 44 comparisons) were included in the meta-analysis. 35 comparisons assessed a single hormone artificial pancreas system, whereas nine comparisons assessed a dual hormone system. Only nine studies were at low risk of bias. Proportion of time in the near normoglycaemic range (3.9-10.0 mmol/L) was significantly higher with artificial pancreas use, both overnight (weighted mean difference 15.15%, 95% confidence interval 12.21% to 18.09%) and over a 24 hour period (9.62%, 7.54% to 11.7%). Artificial pancreas systems had a favourable effect on the proportion of time with sensor glucose level above 10 mmol/L (−8.52%, −11.14% to −5.9%) or below 3.9 mmol/L (−1.49%, −1.86% to −1.11%) over 24 hours, compared with control treatment. Robustness of findings for the primary outcome was verified in sensitivity analyses, by including only trials at low risk of bias (11.64%, 9.1% to 14.18%) or trials under unsupervised, normal living conditions (10.42%, 8.63% to 12.2%). Results were consistent in a subgroup analysis both for single hormone and dual hormone artificial pancreas systems.

**Conclusions:**

Artificial pancreas systems are an efficacious and safe approach for treating outpatients with type 1 diabetes. The main limitations of current research evidence on artificial pancreas systems are related to inconsistency in outcome reporting, small sample size, and short follow-up duration of individual trials.

## Introduction

Despite substantial advances in the treatment of type 1 diabetes, maintaining good glycaemic control without hypoglycaemia remains a challenge for patients at all ages and for healthcare providers. Currently, insulin treatment strategies in type 1 diabetes include either multiple daily insulin injections or continuous subcutaneous insulin infusion with an insulin pump. In 2008, the National Institute for Health and Care Excellence concluded that continuous subcutaneous insulin infusion has a favourable effect on glycated haemoglobin (HbA_1c_) and incidence of hypoglycaemia in patients with type 1 diabetes.^1^ Moreover, a meta-analysis of 19 trials concluded that continuous subcutaneous insulin infusion had a favourable effect on glycaemic control in adults with type 1 diabetes compared with multiple daily insulin injections.^2^ In addition, in a recent cluster randomised controlled trial, patients with type 1 diabetes who used continuous subcutaneous insulin infusion instead of multiple daily insulin injections reported additional benefits in quality of life and greater treatment satisfaction.^3^

Until recently, continuous subcutaneous insulin infusion was mostly guided by self-monitoring of capillary glucose testing.^4^ However, insulin pumps are now also used in conjunction with real time continuous glucose monitoring, hence allowing the patient to manually modify the insulin infusion rate according to continuous glucose monitoring values (known as sensor augmented pump treatment).^4^ ^5^ The recent introduction of a low glucose suspend feature has allowed for automatic pump suspension when a preprogrammed threshold value of continuous glucose monitoring is reached.^6^ Based on a 2016 analysis, the use of sensor augmented pump treatment and the low glucose suspend feature was found to be cost effective compared with continuous subcutaneous insulin infusion and self-monitoring of blood glucose for patients with type 1 diabetes in the United Kingdom.^7^

Artificial pancreas treatment, also referred to as closed loop glucose control, is an emerging treatment option combining an insulin pump and continuous glucose monitoring with a control algorithm to deliver insulin in a glucose responsive manner (that is, a single hormone artificial pancreas system). Glucagon can also be delivered in a similar glucose responsive fashion, as accommodated by dual hormone artificial pancreas systems. Therefore, compared with insulin pumps or sensor augmented pumps, artificial pancreas use can reduce the burden for patients by automatically adjusting the amount of insulin entering the body on the basis of sensor glucose levels. Several artificial pancreas systems have been developed, and their safety and efficacy have been evaluated in many studies, showing promising results. An early pooled analysis included only four studies in an inpatient setting,^8^ whereas an overview published in 2015 summarised existing data from randomised controlled trials up to September 2014.^9^ Finally, a recent meta-analysis summarised evidence from published trials of artificial pancreas systems in outpatients with type 1 diabetes.^10^ Notably, the US Food and Drug Administration has recently approved the first artificial pancreas system for use by people with type 1 diabetes over 14 years of age, based on a safety outpatient study.^11^ This systematic review and meta-analysis aimed to summarise and critically appraise all existing evidence on the clinical efficacy and safety of artificial pancreas systems for the management of type 1 diabetes in the outpatient setting.

## Methods

This systematic review and meta-analysis is based on a prespecified protocol (appendix 1), and is reported according to the preferred reporting items for systematic reviews and meta-analyses (PRISMA) statement (appendix 2).^12^

### Search strategy and selection criteria

We searched Medline, Embase, Cochrane Database of Systematic Reviews, and the Central Register of Controlled Trials from inception to 2 February 2018. Our search strategy was based on search terms describing the intervention (artificial pancreas or closed loop system) in addition to a filter for randomised trials. We omitted terms related to type 1 diabetes to avoid missing potentially relevant studies.^13^ ^14^ We used search terms that had been identified from initial scoping searches, target references, and browsing of database thesauruses (web appendix 3). We imposed no restrictions based on language or publication status, searched ClinicalTrials.gov, and sought for additional studies from snowballing of included records.

We included randomised controlled trials in non-pregnant adults, children, and adolescents with type 1 diabetes in the outpatient setting (including hotels, diabetes camps, or normal living conditions), irrespective of trial design (parallel or crossover) or duration of intervention, which compared artificial pancreas systems with any type of insulin based treatment. Such comparative treatments included multiple daily insulin injections, insulin pump treatment without continuous glucose monitoring or with blinded continuous glucoses monitoring, and sensor augmented pumps with or without a low glucose suspend feature.

### Data extraction

References identified were imported into a reference management software (Endnote, Clarivate Analytics) for deduplication. Potentially eligible records were exported to Covidence (Covidence, Veritas Health Innovation) for screening. Three reviewers (EB, EA, and KK) working independently, screened all records in duplicate, and disagreements were arbitrated by a senior team member (AT). Initially, records were screened at title and abstract level, and potentially eligible studies were assessed in full text.

If multiple records of one study were retrieved, we collated data from all records, and used data from the report with the longest duration of follow-up. We extracted data for study and participant baseline characteristics, interventions, comparators, and clinical outcomes in duplicate (EB, EA, and TK) by using an electronic, pilot tested, data extraction form (web appendix 4). Disagreements were resolved by consensus or following discussion with a senior reviewer (AT).

### Outcomes

The primary outcome was proportion (%) of time when the sensor glucose level was within the near normoglycaemic range (3.9-10 mmol/L). Secondary outcomes included proportion (%) of time when the sensor glucose level was above 10 mmol/L or below 3.9 mmol/L, incidence of severe hypoglycaemia, mean sensor glucose level, total daily insulin needs, and glycated haemoglobin (HbA_1c_). We also used low blood glucose index overnight as an additional outcome to assess hypoglycaemia. Low blood glucose index is a weighted average of the number of hypoglycaemic readings with progressively increasing weights as glucose levels decrease and is associated with the risk of hypoglycaemia and prediction of severe hypoglycaemic episodes.^15^ When available, for proportion (%) of time in the near normoglycaemic range, hyperglycaemia (>10 mmol/L), or hypoglycaemia (<3.9 mmol/L), we extracted data both for 24 hour and overnight periods (as defined in each individual study).

### Statistical analysis

We conducted meta-analyses when data were available for at least two studies. We calculated weighted mean differences with 95% confidence intervals, applying an inverse variance weighted random effects model using the DerSimonian and Laird estimation method.^16^ We also calculated 95% prediction intervals to estimate a predicted range for the true treatment effect in any one individual study.^17^ In addition, to account for uncertainty related to heterogeneity estimates, we calculated 95% confidence intervals applying the Hartung Knapp correction method.^18^ For trials reporting only medians and interquartile ranges, we retrieved mean and variance values from authors of original reports or used appropriate formulas to calculate mean and variance, making no assumption on the distribution of the underlying data.^19^ We combined data both from parallel group and crossover studies. Finally, for crossover studies that reported their results as parallel group trials, we used appropriate methods to impute within patient differences.^20^

We conducted prespecified subgroup analyses based on the mode of use (overnight or over 24 hours) and type of artificial pancreas system (single or dual hormone). A series of a priori decided sensitivity analyses was conducted for the primary outcome, excluding trials at unclear or high risk of bias, trials recruiting people in diabetes camps, or trials with supervised use of artificial pancreas system. We assessed statistical heterogeneity by the χ^2^ based Cochran Q test and the τ^2^ and I^2^ statistics. For HbA_1c_, we synthesised only data from trials with at least eight weeks’ duration per intervention. All analyses were undertaken in RevMan 5.3 (Nordic Cochrane Centre) and Stata 13.0 (Stata Corporation). 

### Assessment of risk of bias in individual studies and across studies

Quality assessment was undertaken in duplicate by two independent reviewers (EB and EA), and disagreements were resolved by consensus or arbitrated by a third reviewer (AT). We used the Cochrane Collaboration risk of bias tool to assess risk of bias for the primary outcome for individual studies. For crossover studies, we also assessed a series of methodological challenges that are related to this specific design (appropriateness of crossover design, carry-over effects, unbiased data).^21^ We used results to provide an evaluation of the overall quality of the included studies (appendix 5) to inform a sensitivity analysis including only trials at low overall risk of bias.

We explored risk of bias across studies, both visually using a contour enhanced funnel plot, and formally using Egger's statistical test.^22^ ^23^ In case of evidence of small study effects, we used the trim and fill method as a sensitivity analysis, to provide an adjusted estimate of the meta-analysis.^24^

### Patient involvement

No patients were involved in definition of the research question or the outcome measures, and interpretation or writing up of results. Data relating to the impact of the intervention on participants’ quality of life were not extracted. Where possible, results of this systematic review and meta-analysis will be disseminated to the patient community or individual patients and families through the investigators of this meta-analysis.

## Results

### Characteristics of included studies

**Fig 1:**
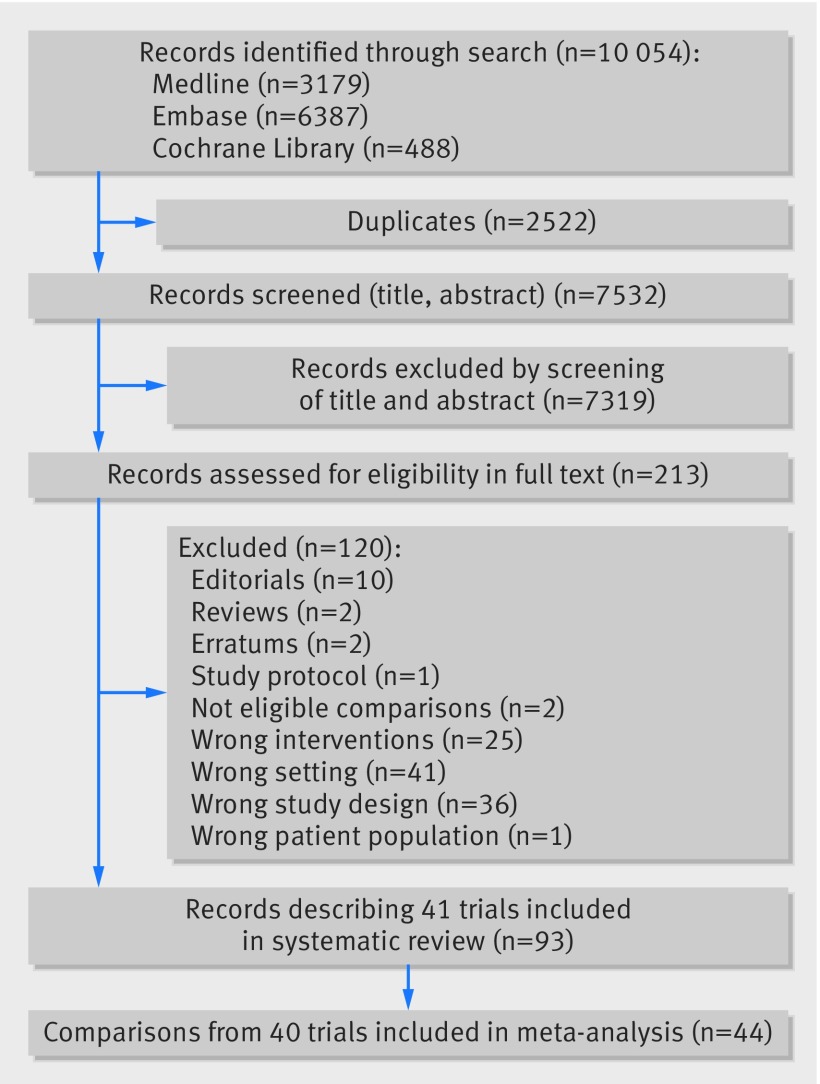
Flow diagram of study selection process

Figure 1 shows the study selection process. Of 10 054 records retrieved, 85 reports qualified for inclusion in our systematic review. After juxtaposing different reports that referred to the same study, 39 publications describing 41 trials (1042 participants with data for 45 comparisons) were used to inform our systematic review.^25^ ^26^ ^27^ ^28^ ^29^ ^30^ ^31^ ^32^ ^33^ ^34^ ^35^ ^36^ ^37^ ^38^ ^39^ ^40^ ^41^ ^42^ ^43^ ^44^ ^45^ ^46^ ^47^ ^48^ ^49^ ^50^ ^51^ ^52^ ^53^ ^54^ ^55^ ^56^ ^57^ ^58^ ^59^ ^60^ ^61^ ^62^ ^63^ One trial did not report data for outcomes assessed and was not included in the meta-analysis.^57^

Table 1 shows characteristics of the 41 studies included in the systematic review and their participants at baseline. The clear majority of included trials used a crossover design,^25^ ^26^ ^27^ ^29^ ^30^ ^31^ ^32^ ^33^ ^34^ ^35^ ^37^ ^38^ ^39^ ^40^ ^41^ ^43^ ^44^ ^45^ ^46^ ^50^ ^51^ ^52^ ^53^ ^54^ ^55^ ^56^ ^57^ ^58^ ^60^ ^61^ ^62^ ^63^ whereas only seven trials were of parallel design.^28^ ^36^ ^42^ ^47^ ^48^ ^49^ ^59^ The duration of 36 trials lasted up to four weeks,^25^ ^26^ ^27^ ^28^ ^29^ ^30^ ^31^ ^32^ ^33^ ^34^ ^35^ ^36^ ^37^ ^38^ ^39^ ^40^ ^41^ ^43^ ^45^ ^46^ ^47^ ^48^ ^49^ ^50^ ^52^ ^53^ ^54^ ^55^ ^56^ ^57^ ^58^ ^59^ ^60^ ^61^ ^62^ whereas the remaining five trials lasted from eight to 30 weeks.^42^ ^44^ ^51^ ^63^ Seventeen trials recruited children or adolescents,^28^ ^30^ ^32^ ^35^ ^36^ ^38^ ^41^ ^49^ ^50^ ^53^ ^54^ ^55^ ^56^ ^57^ ^60^ ^61^ ^63^ 13 recruited adults,^25^ ^27^ ^29^ ^34^ ^37^ ^40^ ^43^ ^44^ ^45^ ^55^ ^62^ ^63^ and 11 recruited a mixed population.^26^ ^31^ ^39^ ^42^ ^46^ ^47^ ^48^ ^51^ ^52^ ^58^ ^59^ The artificial pancreas was used overnight in 16 trials,^29^ ^36^ ^38^ ^39^ ^41^ ^44^ ^46^ ^50^ ^51^ ^52^ ^53^ ^57^ ^58^ ^59^ ^62^ ^63^ and used over 24 hours in the remaining 25 trials.^25^ ^26^ ^27^ ^28^ ^30^ ^31^ ^32^ ^33^ ^34^ ^35^ ^37^ ^40^ ^42^ ^43^ ^45^ ^47^ ^48^ ^49^ ^54^ ^55^ ^56^ ^60^ ^61^ ^63^ In 32 trials, a single hormone artificial pancreas system was assessed (mostly versus unblinded treatment using sensor augmented pump).^25^ ^26^ ^28^ ^29^ ^30^ ^31^ ^32^ ^35^ ^36^ ^37^ ^41^ ^42^ ^43^ ^44^ ^45^ ^46^ ^47^ ^48^ ^49^ ^50^ ^51^ ^52^ ^53^ ^54^ ^57^ ^58^ ^59^ ^60^ ^61^ ^62^ ^63^ Five trials assessed a dual hormone artificial pancreas system, mainly by comparison with insulin pump treatment (consisting of continuous subcutaneous insulin infusion combined with a blinded system of continuous glucose monitoring).^27^ ^34^ ^55^ ^56^ Additionally, four studies evaluated both a single hormone and a dual hormone system against control treatment (as three way crossover trials).^33^ ^38^ ^39^ ^40^

**Table 1:** Baseline characteristics of comparisons included in the systematic review

Study author and year	Trial registration details	Setting	Population	Type of artificial pancreas	Type of comparator	Intervention duration	Length of follow-up*	No of patients
Bally 2017^25^	NCT02727231	Home	Adults	Florence	SAP	24 h	4 weeks	29
Biester 2016^26^	NCT02636491	Home	Adults and adolescents	MD-Logic	SAP	24 h	2 days	10
Blauw 2016^27^	NCT02160275	Home	Adults	Inreda dual hormone CL	Insulin pump treatment	24 h	4 days	10
Breton 2017^28^	NCT02604524	Winter camp	Adolescents	DiAs	SAP	24 h	5 days	32
Brown 2017^29^	NCT02131766, NCT02008188	Hotel or research house	Adults	DiAs	SAP	Overnight	5 days	40
Chernavvsky 2016^30^	NCT01890954	Research house	Adolescents	DiAs USS	Insulin pump treatment	24 h	1 day	16
De Bock 2015^31^	ACTRN12614001005640	Home	Adults and adolescents	Medtronic PID IFB	SAP+LGS	24 h	5 days	8
De Boer 2017^32^	NCT02750267	Hotel or home	Children	DiAs	SAP	24 h	3 days	12
Ekhlaspour 2016a^33^	Not reported	Home	Adults	Single hormone	Insulin pump treatment	24 h	3 days	20
Ekhlaspour 2016b^33^	Not reported	Home	Adults	Dual hormone	Insulin pump treatment	24 h	3 days	20
El-Khatib 2016^34^	NCT02092220	Home	Adults	Dual hormone	Insulin pump treatment or SAP	24 h	11 days	39
Favero 2016^35^	NCT0260878	Diabetes camp	Children	DiAs	SAP	24 h	3 days	30
Forlenza 2017a^37^	NCT02773875	Home	Adults	DiAs	SAP	24 h	2 weeks	19
Forlenza 2017b^36^	NCT02714972	Home	Children and adolescents	Medtronic PHHM	SAP+LGS	Overnight	21 nights	28
Haidar 2015a^38^	NCT02189694	Diabetes camp	Adolescents	Single hormone	Insulin pump treatment	Overnight	3 days	33
Haidar 2015b^38^	NCT02189694	Diabetes camp	Adolescents	Dual hormone	Insulin pump treatment	Overnight	3 days	33
Haidar 2016a^39^	NCT01905020	Home	Adults and adolescents	Single hormone	Insulin pump treatment	Overnight	2 days	28
Haidar 2016b^39^	NCT01905020	Home	Adults and adolescents	Dual hormone	Insulin pump treatment	Overnight	2 days	28
Haidar 2017a^40^	NCT01966393	Home	Adults	Single hormone	SAP	24 h	60 hours	23
Haidar 2017b^40^	NCT01966393	Home	Adults	Dual hormone	SAP	24 h	60 hours	23
Hovorka 2014^41^	NCT01221467	Home	Adolescents	Florence	SAP	Overnight	3 weeks	16
Kingman 2017^42^	Not reported	Outpatient	Adults and adolescents	DiAs	SAP	24 h	5 weeks	37
Kovatchev 2014^43^	NCT01714505, NCT01727817, NCT01742741	Hotel or guesthouse	Adults	DiAs SSM	SAP	24 h	40 hours	20
Kropf 2015^44^	NCT02153190	Home	Adults	DiAs SSM	SAP	Evening and night	8 weeks	32
Leelarantha 2014^45^	NCT01666028	Home	Adults	Florence	SAP	24 h	8 days	17
Ly 2014^46^	NCT01973413	Diabetes camp	Adults and adolescents	DiAs USS	SAP	Overnight	5-6 days	20
Ly 2015a^48^	NCT02366767	Diabetes camp	Adults and adolescents	Medtronic PID IFB	SAP+LGS	24 h	6 days	21
Ly 2015b^47^	Not reported	Diabetes camp	Adults and adolescents	DiAs	SAP	24 h	5 days	16
Ly 2016a^49^	NCT02147860	Diabetes camp	Adolescents	DiAs USS	SAP	24 h	5 days	33
Ly 2016b^50^	Not reported	Diabetes camp	Children and adolescents	Medtronic PID IFB	SAP	Overnight	1 day	21
Nimri 2014^51^	NCT01238406	Home	Adults and adolescents	MD-Logic	SAP	Overnight	6 weeks	24
Nimri 2016^52^	NCT01726829	Home	Children, adolescents and adults	MD-Logic	SAP	Overnight	4 days	75
Phillip 2013^53^	NCT01238406	Diabetes camp	Adolescents	MD-Logic	SAP	Overnight	1 day	54
Renard 2017^54^	Not reported	Outpatient	Children	DiAs	SAP+LGS	24 h	2 days	24
Russell 2014a^55^	NCT01762059	Home and hotel	Adults	Dual hormone	Insulin pump treatment or SAP	24 h	5 days	20
Russell 2014b^55^	NCT01833988	Diabetes camp	Adolescents	Dual hormone	Insulin pump treatment or SAP	24 h	5 days	32
Russell 2016^56^	NCT02105324	Diabetes camp	Preadolescents	Dual hormone	Insulin pump treatment or SAP	24 h	5 days	19
Schierloh 2015^57^†	Not reported	Home	Children	Florence	SAP	Overnight	4 days	15
Sharifi 2016^58^	Not reported	Home	Adults and adolescents	Medtronic PID IFB	SAP+LGS	Overnight	4 days	28
Spaic 2017^59^	NCT02438189	Home	Adults and adolescents	Medtronic PHHM	SAP+LGS	Overnight	21 nights	30
Tauschmann 2016a^61^	NCT01873066	Home	Adolescents	Florence	SAP	24 h	7 days	12
Tauschmann 2016b^60^	NCT01873066	Home	Adolescents	Florence	SAP	24 h	3 weeks	12
Thabit 2014^62^	NCT01440140	Home	Adults	Florence	SAP	Overnight	4 weeks	24
Thabit 2015a^63^	NCT01961622	Home	Adults	Florence	SAP	24 h	12 weeks	33
Thabit 2015b^63^	NCT01778348	Home	Children and adolescents	Florence	SAP	Overnight	12 weeks	25

DiAs=Diabetes Assistant; USS=Unified Safety System; SAP=sensor augmented pump treatment; MPC=model predictive control; PID=proportional integral derivative; IFB=insulin feedback; LGS=low glucose suspend; PHHM=predictive hyperglycaemia and hypoglycaemia minimisation; SSM=safety supervision module.

* For crossover trials, length of follow-up refers to the duration of each period, excluding washout period.

† Not included in the meta-analysis.

In six studies assessing sensor augmented pump treatment, control treatment comprised a sensor augmented pump combined with an low glucose suspend feature.^31^ ^36^ ^48^ ^54^ ^58^ ^59^ Among trials evaluating single hormone artificial pancreas systems, 13 used the DiAs platform,^28^ ^29^ ^30^ ^32^ ^35^ ^37^ ^42^ ^43^ ^44^ ^46^ ^47^ ^49^ ^54^ eight used the Florence implementation,^25^ ^41^ ^45^ ^57^ ^60^ ^61^ ^62^ ^63^ four used the MD-Logic platform,^26^ ^51^ ^52^ ^53^ and six used a Medtronic device.^31^ ^36^ ^48^ ^50^ ^58^ ^59^ Most trials used a model predictive control algorithm,^25^ ^29^ ^34^ ^35^ ^37^ ^38^ ^39^ ^40^ ^41^ ^43^ ^44^ ^45^ ^54^ ^55^ ^56^ ^57^ ^60^ ^61^ ^62^ ^63^ five used a proportional integral derivative algorithm,^27^ ^31^ ^48^ ^50^ ^58^ four used a fuzzy logic algorithm,^26^ ^51^ ^52^ ^53^ four used a control to range algorithm,^30^ ^32^ ^46^ ^49^ and the remainder used other algorithms or did not provide relevant details.^28^ ^33^ ^36^ ^42^ ^47^ ^59^ Twenty one comparisons used a Dexcom sensor for continuous glucose monitoring,^28^ ^29^ ^30^ ^32^ ^34^ ^35^ ^37^ ^38^ ^40^ ^42^ ^43^ ^44^ ^46^ ^47^ ^49^ ^54^ ^55^ ^56^ while 12^27^ ^31^ ^36^ ^39^ ^48^ ^50^ ^51^ ^52^ ^53^ ^58^ ^59^ and nine^25^ ^41^ ^45^ ^57^ ^60^ ^61^ ^62^ ^63^ comparisons used an Enlite Sensor or a FreeStyle Navigator in the artificial pancreas systems, respectively. Type of sensor for continuous glucose monitoring was not reported in two trials.^26^ ^33^ In 41 comparisons, the type of sensor for continuous glucose monitoring was identical between artificial pancreas and control arms, whereas four trials did not report information for type of sensor used in the control arm.^26^ ^30^ ^47^ ^49^

In terms of setting, 13 trials were held in a diabetes camp or a guesthouse,^28^ ^29^ ^35^ ^38^ ^43^ ^46^ ^47^ ^48^ ^49^ ^50^ ^53^ ^55^ ^56^ and in 26 trials, participants were at home.^25^ ^26^ ^27^ ^30^ ^31^ ^32^ ^33^ ^34^ ^36^ ^37^ ^39^ ^40^ ^41^ ^44^ ^45^ ^51^ ^52^ ^55^ ^57^ ^58^ ^59^ ^60^ ^61^ ^62^ ^63^ Only in a small subset of trials were participants using artificial pancreas unsupervised under normal living conditions^25^ ^26^ ^41^ ^45^ ^60^ ^61^ ^62^ ^63^; the remaining studies either used remote monitoring or did not provide relevant details. Participants’ mean age and HbA_1c_ at baseline ranged from 7.0 to 47.0 years and from 6.9% to 8.6%, respectively.

### Risk of bias assessment results

Risk of bias assessment for the primary outcome is presented in appendices 6 and 7. Only nine studies were at low risk of bias. Most studies were deemed at high risk for bias, because either they reported median values instead of mean values, or reported results that required extensive use of imputation methods to be used in meta-analyses.

### Primary outcome

**Fig 2:**
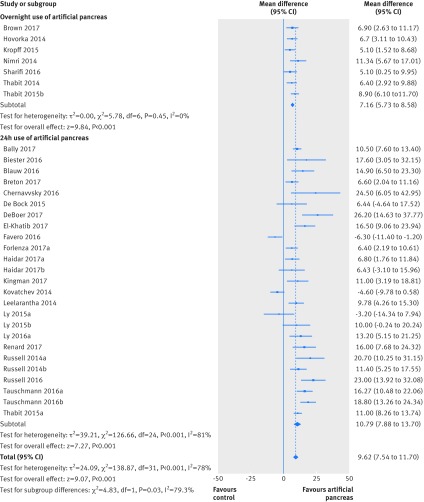
Weighted mean difference in proportion (%) of 24 hour period in near normoglycaemic range (glucose concentration 3.9-10.0 mmol/L), artificial pancreas use versus control treatment

All meta-analysis results are presented as summary effect estimates for artificial pancreas systems versus control treatment. Compared with control treatment, use of artificial pancreas was associated with an increased percentage of time (140 additional minutes) in near normoglycaemia (3.9-10.0 mmol/L) over 24 hours (overall weighted mean difference 9.62% (95% confidence interval 7.54% to 11.7%); I^2^=78%, τ^2^=24.09, 32 studies). This effect was consistent both for trials using artificial pancreas overnight (7.16% (5.73% to 8.58%); 0%, 0.0, seven studies) or over 24 hours (10.79% (7.88% to 13.7%); 81%, 39.21, 25 studies; fig 2). The confidence interval for the overall effect estimate after applying the Hartung Knapp correction was 7.83% to 12.41%, whereas the 95% prediction interval was −0.63% to 19.87%. Of note, the 95% prediction interval was above zero when the artificial pancreas was used overnight (5.29% to 9.02%), suggesting that artificial pancreas use will be beneficial in at least 95% of the individual study settings when applied overnight. However, the prediction interval contained negative values when applied over 24 hours (−2.52% to 24.1%), and therefore might not be beneficial in some settings.

**Fig 3:**
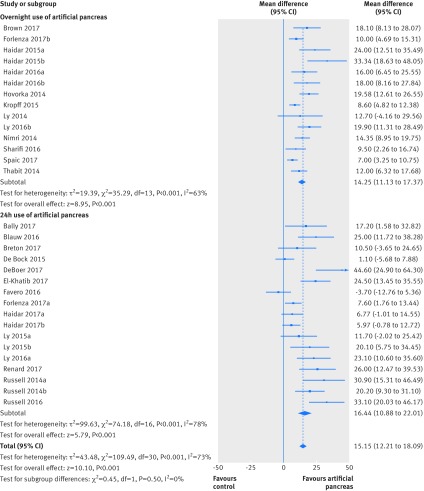
Weighted mean difference in proportion (%) of overnight period in near normoglycaemic range (glucose concentration 3.9-10.0 mmol/L), artificial pancreas use versus control treatment

The favourable effect of artificial pancreas use over control treatment was more evident on the proportion of time in near normoglycaemia overnight (overall weighted mean difference 15.15% (95% confidence interval 12.21% to 18.09%); I^2^=73%, τ^2^=43.48, 31 studies). This effect was consistent when artificial pancreas was used either only overnight (14.25% (11.13% to 17.37%); 63%, 19.39, 14 studies) or over 24 hours (16.44% (10.88% to 22.01%); 78%, 99.63, 17 studies; fig 3), even when the Hartung Knapp correction was applied (appendix 13). Respective 95% prediction intervals suggested that effect on time in near normoglycaemia overnight would be beneficial in at least 95% of the individual study settings when artificial pancreas was applied overnight (4.04% to 24.45%), but not when applied over 24 hours (−5.68% to 38.56%).

### Secondary outcomes

**Fig 4:**
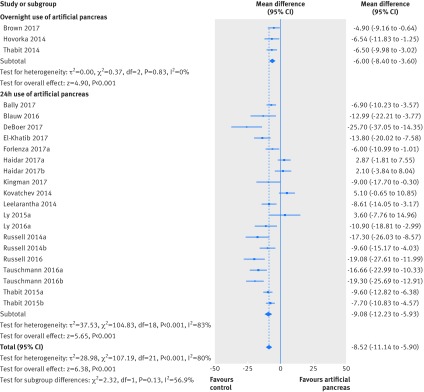
Weighted mean difference in proportion (%) of 24 hour period in hyperglycaemia (glucose concentration >10.0 mmol/L), artificial pancreas use versus control treatment

Use of artificial pancreas had a favourable effect on time in hyperglycaemia (glucose concentrations >10 mmol/L) during the entire day. Compared with control treatment, this period was shortened by about two hours (overall weighted mean difference −8.52% (95% confidence interval −11.14% to −5.9%); I^2^=80%, τ^2^=28.98, 22 studies), both in trials using artificial pancreas overnight (−6.0% (−8.4% to −3.6%); 0%, 0.0, three studies) and those using artificial pancreas over 24 hours (−9.08% (−12.23% to −5.93%); 83%, 37.53, 19 studies; fig 4). Similarly, the time when glucose concentrations were higher than 10.0 mmol/L overnight was also shortened compared with control treatment (−11.12% (−13.8% to −8.44%); 71%, 26.13, 23 studies), both in trials that used artificial pancreas either only overnight (−9.23% (−11.67% to −6.79%); 51%, 8.26, 12 studies) or over 24 hours (−13.86% (−19.83% to −7.9%); 80%, 77.07, 11 studies; appendix 8).

**Fig 5:**
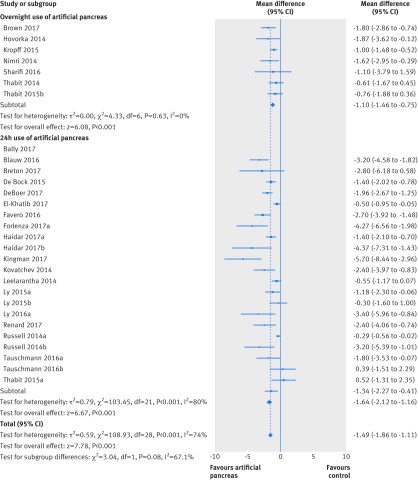
Weighted mean difference in proportion (%) of 24 hour period with glucose concentrations lower than 3.9 mmol/L, artificial pancreas use versus control treatment

Time when glucose concentrations were lower than 3.9 mmol/L over a 24 hour period was shortened with artificial pancreas use by about 20 minutes (overall weighted mean difference −1.49% (95% confidence interval −1.86% to −1.11%); I^2^=74%, τ^2^=0.59, 29 studies), compared with control treatment (fig 5). Results were consistent for overnight time when concentrations were lower than 3.9 mmol/L (−2.22% (−2.78% to −1.65%); 72%, 1.34, 29 studies; appendix 9). Data on incidence of severe hypoglycaemia (that is, hypoglycaemia requiring third party assistance) were available in 27 studies (804 patients). Overall, incidence of severe hypoglycaemia was very low both in groups using artificial pancreas (six episodes) and control treatment (three episodes). Use of artificial pancreas was also associated with a reduction in overnight low blood glucose index (−0.37 (−0.56 to −0.18); 85%, 0.06, 11 studies).

**Fig 6:**
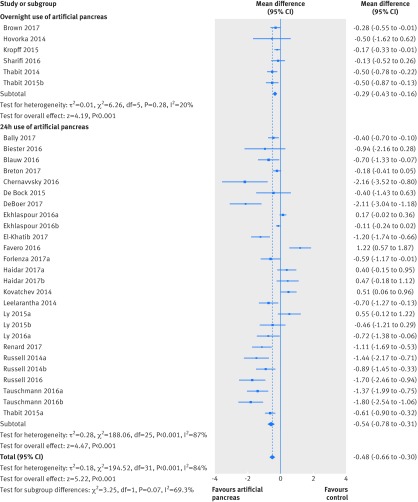
Weighted mean difference in mean levels of sensor blood glucose (mmol/L) over 24 hours, artificial pancreas use versus control treatment

**Fig 7:**
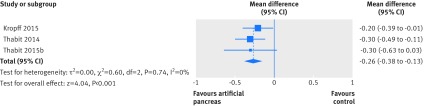
Weighted mean difference in change in HbA_1c_ (%), artificial pancreas use versus control treatment

Compared with control treatment, use of artificial pancreas had a favourable effect on mean levels of sensor blood glucose over 24 hours, which fell by 0.48 mmol/L (95% confidence interval 0.3 to 0.66; I^2^=84%, τ^2^=0.18, 32 studies; fig 6). Results were more favourable for mean levels of sensor blood glucose overnight (overall weighted mean difference −0.81 mmol/L (−1.03 to −0.6); 78%, 0.3, 35 studies; appendix 10). These findings were consistent with the effect of artificial pancreas use on HbA_1c_ (−0.26% (−0.38% to −0.13%); 0%, 0.0, three studies; fig 7). Finally, no difference between artificial pancreas use and control treatment was seen in the mean daily needs for insulin (−0.21 IU (−1.64 to 1.22); 77%, 4.45, 14 studies; appendix 11). Appendix 12 presents 95% Hartung Knapp confidence intervals and prediction intervals for all outcomes.

### Sensitivity and subgroup analyses

**Fig 8:**
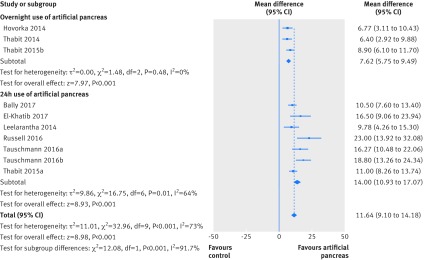
Weighted mean difference in proportion (%) of 24 hour period in near normoglycaemic range (glucose concentration 3.9-10.0 mmol/L), artificial pancreas use versus control treatment. Sensitivity analysis includes only trials at low risk of bias

**Fig 9:**
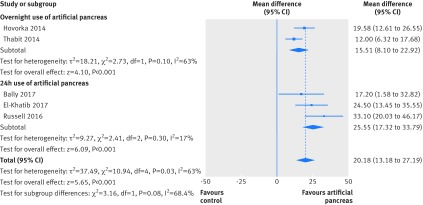
Weighted mean difference in proportion (%) of overnight period in near normoglycaemic range (glucose concentration 3.9-10.0 mmol/L), artificial pancreas use versus control treatment. Sensitivity analysis includes only trials at low risk of bias

Results for the proportion of time in near normoglycaemia were similar in a sensitivity analysis including only trials at low risk of bias, both over 24 hours (overall weighted mean difference 11.64% (95% confidence interval 9.1% to 14.18%); 10 studies) and overnight (20.18% (13.18% to 27.19%); five studies; fig 8 and fig 9). Similarly, results for near normoglycaemia did not differ in a series of sensitivity analyses excluding trials that using artificial pancreas in diabetes camps or including only trials using artificial pancreas in unsupervised patients in normal living conditions. This similarity was seen both for the 24 hour period (10.42% (95% confidence interval 8.63% to 12.2%) and 10.67% (8.33% to 13.01%), respectively; appendices 13 and 14) and overnight period (13.47% (10.41% to 16.54%) and 15.53% (10.12% to 20.94%), respectively; appendices 15 and 16).

We also did a post hoc sensitivity analysis excluding trials comparing artificial pancreas systems with low glucose suspend systems, to explore their effect on hypoglycaemia. Time when concentrations were lower than 3.9 mmol/L was shortened compared with control treatment (overall weighted mean difference −1.59% (95% confidence interval −1.99% to −1.19%) for 24 hour period, −2.53% (−3.18% to −1.87%) for overnight period; appendices 17 and 18). Finally, for all outcomes, results were consistent with those of the main analysis in a prespecified subgroup analysis based on type of artificial pancreas used (that is, single hormone versus dual hormone artificial pancreas; table 2).

**Table 2:** Summary of subgroup meta-analyses results based on type of artificial pancreas system used (single hormone or dual hormone)

Outcome and time period	No of studies (single/dual hormone)	Artificial pancreas system *v* control treatment* (weighted mean difference (95% CI), I^2^, τ^2^)	
		Single hormone	Dual hormone
Proportion (%) of time in near normoglycaemia (glucose concentration 3.9-10.0 mmol/L)			
24 h	26/6	8.53 (6.34 to 10.72), 78%, 22.00	15.16 (10.68 to 19.63), 43%, 13.08
Overnight	23/8	12.77 (9.82 to 15.71), 68%, 29.73	22.84 (15.08 to 30.60), 74%, 88.82
Proportion (%) of time with glucose concentration greater than >10.0 mmol/L			
24 h	16/6	−7.52 (−10.38 to −4.66), 80%, 24.96	−11.58 (−18.17 to −4.99), 81%, 36.43
Overnight	15/8	−8.4 (−10.22 to −6.58), 24%, 2.82	−17.21 (−25.58 to −8.85), 87%, 121.35
Proportion (%) of time with glucose concentration lower than 3.9 mmol/L			
24 h	24/5	−1.28 (−1.65 to −0.92), 72%, 0.45	−2.95 (−4.03 to −1.87), 30%, 0.45
Overnight	24/7	−1.82 (−2.38 to −1.27), 70%, 1.00	−4.04 (−5.59 to −2.48), 47%, 1.93
Low blood glucose index, overnight	11/0	−0.37 (−0.56 to −0.18), 85%, 0.06	Not estimable
Mean sensor glucose value (mmol/L)			
24 h	25/7	−0.41 (−0.61 to −0.20), 83%, 0.19	−0.76 (−1.31 to −0.22), 89%, 0.45
Overnight	29/8	−0.67 (−0.89 to −0.45), 76%, 0.24	−1.47 (−2.14 to −0.79), 80%, 0.72
Daily insulin need (IU), over 24 h	13/1	−0.47 (−1.84 to 0.89), 76%, 3.78	Not estimable

* Studies with single hormone systems mainly used sensor augmented pump treatment as a comparator; those with dual hormone systems mainly used insulin pump treatment as a comparator.

### Small study effects

Both visually and formally, no evidence of small study effects was seen for the proportion of time in near normoglycaemia over 24 hours (P=0.129). However, evidence of small study effects was seen (P<0.001) for the proportion of time in near normoglycaemia overnight, and visual inspection of the contour enhanced funnel plot suggested that small negative studies were missing (appendix 19). Nevertheless, the adjusted meta-analytical estimate after use of the trim and fill method remained in favour of artificial pancreas use (weighted mean difference 10.39% (95% confidence interval 7.30% to 13.49%), P<0.001).

## Discussion

### Key findings

Our data suggest that use of artificial pancreas is associated with almost two and a half additional hours in near normoglycaemia over a 24 hour period compared with control treatment, mainly due to its favourable effect during the overnight period. This finding was also verified by its effect on time in hyperglycaemia (two hours less than control treatment) and in hypoglycaemia (20 minutes less). Results were robust both for single and dual hormone systems, and were consistent in all sensitivity analyses performed—including an analysis restricted to trials under normal living conditions without remote monitoring, supporting the convenience and ease of use of artificial pancreas systems. 

Finally, this favourable effect was also evident in the relative reduction of mean blood glucose levels by 0.48 mmol/L, which is consistent with the HbA_1c_ reduction of about 0.3% recorded in trials with a duration of more than eight weeks per intervention.^44^ ^63^ ^64^ Overall, our results reflect the progress made over recent decades of extensive research and development in artificial pancreas use.

### Comparison with other studies

Despite heterogeneity in interventions and comparators used, our systematic review provides a valid and up to date overview on the use of artificial pancreas. An early pooled analysis of randomised controlled trials with artificial pancreas systems, published in 2011, included only four studies in an inpatient setting.^8^ The effect of artificial pancreas in the outpatient setting was examined in a recent systematic review and meta-analysis of 24 randomised controlled trials (585 participants).^10^ However, validity and clinical interpretation potential of the results were undermined by methodological decisions regarding the definition of outcomes, handling of median values, and exclusion of evidence from grey literature sources, potentially missing a substantial amount of evidence.^65^ Our systematic review and meta-analysis incorporated a much larger pool of eligible studies (n=41) and participants (n=1042) and assessed a broader variety of outcomes, focusing on outcome definitions considered most important in trials evaluating artificial pancreas systems.^66^ ^67^ ^68^

Furthermore, Weisman and colleagues analysed only 24 hour outcomes for studies investigating artificial pancreas use for 24 hour periods and analysed only overnight outcomes for studies investigating artificial pancreas use overnight, even when individual trials provided data for both periods.^10^ Instead, our systematic review dealt with the research question by conducting separate analyses based on all four combinations of outcome assessment period (24 hours or overnight) and duration of intervention use (for 24 hours or solely overnight).

### Strengths and limitations of study

Composition of the review team ensured appropriate methodological and field expertise, but also access to additional study data from individual studies.^41^ ^45^ ^60^ ^61^ ^62^ ^63^ To ensure internal validity of our conclusions, we implemented current guidelines for the conduct and reporting of systematic reviews,^12^ and adhered to a prespecified protocol with minimal deviations. We undertook a comprehensive search of multiple databases without imposing any restrictions based on language or publication type, and assessed quality of trials using valid methodological tools. Moreover, we synthesised existing data using appropriate methodology to account for inappropriate reporting and analysis methods used in some of the trials included. In addition, we conducted a range of sensitivity analyses excluding trials using remote monitoring or trials at high risk of bias, to examine clinical relevance and robustness of our findings.

We acknowledge several limitations at the evidence and review level. Most trials had a small sample size, limiting the precision of our effect estimates. Despite using broad inclusion criteria, existing studies provided limited insight regarding clinically relevant subgroups, such as those with increased hypoglycaemia burden, hypoglycaemia unawareness, gastroparesis, blindness, high HbA_1c_, treated with corticosteroids, or from ethnic minorities.^69^ Many trials were at high or unclear risk of bias owing to suboptimal reporting. Specifically, most trials reported effect estimates for outcomes related to hypoglycaemia using median values and interquartile ranges, thus we had to impute mean and standard deviation values for the meta-analyses. In addition, several crossover trials reported results as parallel group studies,^47^ ^48^ ^49^ which also required use of imputation methods to allow synthesis of results. 

Furthermore, we did not register our protocol at a publicly available database, and submitted it only for internal peer review. We focused on surrogate outcomes and did not extract evidence for specific patient outcomes such as quality of life, incidence of ketoacidosis, or catheter occlusion. Instead, we adopted a practical approach focusing on outcomes that we expected to be most and best reported in trials.^68^ Moreover, for missing or inappropriately reported data, we refrained from contacting study authors other than those who were members of the review group, but used appropriate methodology to impute data.^70^

Finally, most analyses had a high degree of heterogeneity, which could be attributed to differences in continuous glucose monitoring used, sensor accuracy and performance, compliance with artificial pancreas use in supervised and unsupervised settings, and comparators used during control treatment if sensor glucose readings were or were not available. These differences could explain wide prediction intervals that included zero values for most outcomes in trials using artificial pancreas over 24 hours; thus, related findings should be interpreted with caution. By contrast, strong evidence indicated that overnight use of artificial pancreas would be beneficial for outcomes regarding time in near normoglycaemia, hyperglycaemia, or hypoglycaemia (95% prediction intervals excluding zero values), suggesting that this treatment effect can be expected in future patients.

### Implications

Our study highlights some pitfalls in the conduct and reporting of artificial pancreas trials. Many trials had a short duration or were designed to assess the feasibility or safety, rather than long term effectiveness. Despite existing guidance, we noted significant variation in outcomes assessed and metrics used.^71^ Research groups should report a minimum set of agreed outcome measures and respective metrics.^66^ ^67^ ^68^ To ensure the clinical relevance and feasibility of this core outcome set, it is crucial that its development involves all key stakeholders, including patients, their families, clinicians, researchers, statisticians, methodologists, industry representatives, regulatory authorities, and funders. 

To maximise the yield of information and to facilitate analysis and synthesis of evidence overall, the use of a common repository for data on individual patients could be agreed on.^72^ ^73^ Such repositories would facilitate free dissemination of raw trial data, allowing for replication of previous research findings using various analysis approaches (for example, a repeated measures analysis) of clinically relevant outcomes. Moreover, to enhance the external validity of evidence, future trials should broaden inclusion criteria and recruit more heterogeneous populations, including ethnic minorities.

The performance of current artificial pancreas systems could be enhanced by the optimisation of system components. Use of novel insulin analogues with faster pharmacokinetics,^74^ the development of glucagon preparation stable at room temperature, and integration of artificial pancreas components into one device could further enhance user experience and artificial pancreas usefulness, and thus increase uptake. Future research should explore the potential differences between individual components (algorithms, continuous glucose monitoring) and determine their clinical relevance. 

Upcoming trials should clarify the differences between single hormone and dual hormone systems, and explore artificial pancreas use in relevant groups of people with type 2 diabetes such as those with inpatient hyperglycaemia.^75^ Moreover, the effect of artificial pancreas use on quality of life and on reducing patient burden should be further explored,^76^ considering that patients with type 1 diabetes and their carers have shown a positive attitude towards artificial pancreas systems.^77^ ^78^ ^79^ Finally, to support adoption, cost effectiveness should be assessed to allow for reimbursement by various healthcare systems, and ensure that adequate infrastructure exists.

### Conclusions

Our systematic review and meta-analysis has shown that artificial pancreas systems are an efficacious and safe treatment approach for people with type 1 diabetes, leading to increased time in near normoglycaemic range, and reduced time in hypoglycaemia and hyperglycaemia. The results were verified for all types of artificial pancreas and in all sensitivity analyses. Further research with rigorous studies, cooperation of research groups in terms of outcome reporting, and cost effectiveness data are required to verify these findings and support adoption of artificial pancreas systems in clinical practice.

**What is already known on this topic**

Individual studies have shown artificial pancreas use to be safe and efficacious in inpatients, patients under close monitoring, and outpatients with type 1 diabetes
The US Food and Drug Administration recently approved artificial pancreas use for patients aged 14 years and older with type 1 diabetes
Previous meta-analyses on artificial pancreas systems have provided limited findings, mainly owing to the low number of studies incorporated and heterogeneous definitions of outcomes

**What this study adds**

In view of all the available evidence from randomised controlled trials, artificial pancreas treatment significantly improves glycaemic control while reducing the burden of hypoglycaemia in outpatients with type 1 diabetes
Results are consistent for people using artificial pancreas systems unsupervised under normal living conditions, and for both single hormone and dual hormone systems
The current research evidence on artificial pancreas systems is limited by inconsistency in outcome reporting, small sample size, and short follow-up duration of individual trials

## Web extra

Extra material supplied by authors

**Web appendix:** Appendices

## Notes

Contributors: EB, HT, and AT conceived and designed the study. EB and EA did the scientific literature search. EB, KK, EA, and AT did literature screening. EB, EA, TK, and AT extracted data. EB, EA, and AT did quality assessment of the included studies. EB, TK, A-BH, RH, and AT did the analyses. EB, KK, HT, MT, TK, RH, and AT wrote the first draft of the report. All authors contributed to interpretation and edited the draft report. AT is the study guarantor, had full access to all the trial level data in the study, takes responsibility for the integrity of the data, and accuracy of the data analysis, and had the final responsibility to submit for publication.

Ethical approval: Ethical approval not required.

Data sharing: No additional data available.

The lead author affirms that the manuscript is an honest, accurate, and transparent account of the study being reported; that no important aspects of the study have been omitted; and that any discrepancies from the study as planned have been explained.
