# Supplementary material for: Artificial pancreas treatment for outpatients with type 1 diabetes: systematic review and meta-analysis
Source: BMJ. 2018 Apr 18;361:k1310. doi: 10.1136/bmj.k1310 (PMC5902803; doi:10.1136/bmj.k1310)
Supplement: Supplementary file 1 — Web appendix: Appendices [file beke039000.ww.pdf]

Appendices

Appendix 1

Protocol

Closed-loop insulin therapy for type 1 diabetes: a systematic review and meta-analysis

Inclusion and exclusion criteria

Population

- Non-pregnant adults and children with type 1 diabetes, as defined in each individual study that were assessed in an outpatient setting (including hotel and diabetes camp settings) or under free-living conditions in their home and work environment.

Intervention

- Any closed-loop delivery system, defined as a system utilising a control algorithm, which autonomously increases and decreases insulin delivery based on real-time sensor glucose concentrations, assessed either during daytime, overnight period, or the day-and-night period.

Comparators

- Any type of insulin based therapy, including multiple daily injections (MDI), insulin pump therapy, sensor-augmented insulin pump therapy, sensor-augmented insulin pump with a low glucose suspend (LGS) feature.

Outcomes

Primary outcome:

Proportion of time that glucose level was within the near normoglycaemic range (3.9 - 10 mmol/l) (both overnight, and during a 24h period).

Secondary outcomes:

- % of time during day and night (24h) or night only that glucose level was below 3.9 mmol/l
- % of time during day and night (24h) or night only that glucose level was above 10 mmol/l
- area under the curve (AUC) of glucose < 3.5 mmol/l
- low blood glucose index (LBGI)
- Mean blood glucose levels
- HbA_{1c}
- Insulin amount administered

Study design

Randomised controlled trials, with parallel group or cross-over design, irrespective of duration of intervention.

Information sources

Search strategy

Search strategy based only on the intervention (Closed-loop system) and a filter for randomised trials, to avoid missing potentially relevant studies, as recommended in the Centre for Reviews and Dissemination (CRD)

guidance for undertaking reviews in health care and the Cochrane Handbook. We will use search terms that have been identified from initial scoping searches, target references and browsing of database thesauri (i.e. Medline MeSH and Embase Emtree). We have developed search strategies specifically for each database based on the search features and controlled vocabulary of every individual bibliographic database. We will search the following databases and resources (via relevant interfaces):

- MEDLINE (PubMed)
- EMBASE (OvidSP)
- Cochrane Database of Systematic Reviews (CDSR) (Wiley Online Library)
- Cochrane Central Register of Controlled Trials (CENTRAL) (Wiley Online Library)

We will also look for completed and on-going trials by searching the NIH ClinicalTrials.gov (http://www.clinicaltrials.gov/) trial registry.

We will impose no restrictions based on language or publication status. References identified will be imported in Endnote reference management software for de-duplication. Finally, we will export potentially eligible records to Covidence™ for further handling (screening and data extraction).

Study selection & data collection

All records will be screened via Covidence™, by two reviewers, working independently, and disagreements will be arbitrated by a senior team member. Initially, records will be screened at title and abstract level. Full texts for potentially eligible studies will be imported into Covidence™ and screened as described previously. Finally, we will extract data for the following variables: study and participant baseline characteristics, details for the interventions (i.e. single-hormone, algorithm utilised) and comparators, and clinical outcomes. Data will be extracted by two reviewers, using a piloted, data extraction form. Disagreements will be resolved by consensus or following discussion with a senior reviewer. For crossover studies that report their results as parallel group trials, we will use appropriate methodology to impute within-patient differences.

Study quality assessment

We will assess the methodological quality of included RCTs using the Cochrane Risk of Bias Tool. For crossover studies we will use a modified version to assess a series of methodological challenges that are linked with this specific design. We will use results for descriptive purposes to provide an evaluation of the overall quality of the included studies, but also to inform a sensitivity analysis. Quality assessment will be undertaken by two independent reviewers, and disagreements will be resolved by consensus or arbitrated by a third reviewer.

Data synthesis

Methods of analysis

We will combine data both from parallel group and cross-over studies if appropriate. We will calculate mean differences with 95% confidence intervals, using an inverse-variance weighted random effects model.

Subgroup analyses

Depending on accrued evidence, for the primary outcome we plan to conduct subgroup analyses based on mode of intervention (overnight or 24h use of closed-loop delivery system), and type of closed-loop (single vs dual-hormone closed-loop).

Sensitivity analyses

We will do sensitivity analysis for the primary outcome excluding trials at unclear or high risk of bias, trials conducted at other settings than home or hotel, and supervised trials.

Investigation of heterogeneity

We will assess presence of statistical heterogeneity by means of the chi-square-based Cochran Q test and the magnitude of heterogeneity by means of the I^2 statistic, with P values < 0.10 and I^2 > 50% respectively representing high heterogeneity. All analyses will be undertaken in Revman.

This protocol was submitted as a module assignment for the Systematic Review module for an MSc on Medical Research Methodology at Aristotle University Thessaloniki, and internally peer reviewed.

Appendix 2: PRISMA statement

Section/topic	#	Checklist item	Reported on page #
**TITLE**			
Title	1	Identify the report as a systematic review, meta-analysis, or both.	1
**ABSTRACT**			
Structured summary	2	Provide a structured summary including, as applicable: background; objectives; data sources; study eligibility criteria, participants, and interventions; study appraisal and synthesis methods; results; limitations; conclusions and implications of key findings; systematic review registration number.	2
**INTRODUCTION**			
Rationale	3	Describe the rationale for the review in the context of what is already known.	3
Objectives	4	Provide an explicit statement of questions being addressed with reference to participants, interventions, comparisons, outcomes, and study design (PICOS).	3
**METHODS**			
Protocol and registration	5	Indicate if a review protocol exists, if and where it can be accessed (e.g., Web address), and, if available, provide registration information including registration number.	3, appendix 1
Eligibility criteria	6	Specify study characteristics (e.g., PICOS, length of follow-up) and report characteristics (e.g., years considered, language, publication status) used as criteria for eligibility, giving rationale.	4
Information sources	7	Describe all information sources (e.g., databases with dates of coverage, contact with study authors to identify additional studies) in the search and date last searched.	3, 4

Search	8	Present full electronic search strategy for at least one database, including any limits used, such that it could be repeated.	Appendix 3
Study selection	9	State the process for selecting studies (i.e., screening, eligibility, included in systematic review, and, if applicable, included in the meta-analysis).	4
Data collection process	10	Describe method of data extraction from reports (e.g., piloted forms, independently, in duplicate) and any processes for obtaining and confirming data from investigators.	4, appendix 4
Data items	11	List and define all variables for which data were sought (e.g., PICOS, funding sources) and any assumptions and simplifications made.	4
Risk of bias in individual studies	12	Describe methods used for assessing risk of bias of individual studies (including specification of whether this was done at the study or outcome level), and how this information is to be used in any data synthesis.	5, appendix 5
Summary measures	13	State the principal summary measures (e.g., risk ratio, difference in means).	4
Synthesis of results	14	Describe the methods of handling data and combining results of studies, if done, including measures of consistency (e.g., I^2) for each meta-analysis.	4, 5

**Section/topic**	**#**	**Checklist item**	**Reported on page #**
Risk of bias across studies	15	Specify any assessment of risk of bias that may affect the cumulative evidence (e.g., publication bias, selective reporting within studies).	5
Additional analyses	16	Describe methods of additional analyses (e.g., sensitivity or subgroup analyses, meta-regression), if done, indicating which were pre-specified.	4-5
**RESULTS**			

Study selection	17	Give numbers of studies screened, assessed for eligibility, and included in the review, with reasons for exclusions at each stage, ideally with a flow diagram.	5, Figure 1
Study characteristics	18	For each study, present characteristics for which data were extracted (e.g., study size, PICOS, follow-up period) and provide the citations.	Table 1
Risk of bias within studies	19	Present data on risk of bias of each study and, if available, any outcome level assessment (see item 12).	6, appendices 6-7
Results of individual studies	20	For all outcomes considered (benefits or harms), present, for each study: (a) simple summary data for each intervention group (b) effect estimates and confidence intervals, ideally with a forest plot.	6-8, Figures 2-7, appendices 8-12
Synthesis of results	21	Present results of each meta-analysis done, including confidence intervals and measures of consistency.	6-8 Figures 2-7, appendices 8-12
Risk of bias across studies	22	Present results of any assessment of risk of bias across studies (see Item 15).	8, appendix 19
Additional analysis	23	Give results of additional analyses, if done (e.g., sensitivity or subgroup analyses, meta-regression [see Item 16]).	7-8, Table 2, appendices 13-18
**DISCUSSION**			
Summary of evidence	24	Summarize the main findings including the strength of evidence for each main outcome; consider their relevance to key groups (e.g., healthcare providers, users, and policy makers).	8
Limitations	25	Discuss limitations at study and outcome level (e.g., risk of bias), and at review-level (e.g., incomplete retrieval of identified research, reporting bias).	8-9
Conclusions	26	Provide a general interpretation of the results in the context of other evidence, and implications for future	10

		research.	
**FUNDING**			
Funding	27	Describe sources of funding for the systematic review and other support (e.g., supply of data); role of funders for the systematic review.	10

Appendix 3

Search strategy

Embase (OvidSP)

#1. Artificial pancreas.mp. or exp artificial pancreas/
#2. exp bioartificial organ/
#3. (pancreas or insulin or diabet*).mp.
#4. 2 and 3
#5. exp bionics/
#6. 3 and 5
#7. bionic pancreas.mp.
#8. synthetic pancreas.mp
#9. artificial endocrine pancreas.mp.
#10. artificial beta cell*.mp.
#11. artificial b cell*.mp.
#12. artificial b-cell*.mp.
#13. closed-loop*.mp.
#14. 3 and 13
#15. closed loop*.mp.
#16. 3 and 15
#17. bioartificial pancreas.mp.
#18. bio-artificial pancreas.mp.
#19. 1 or 4 or 6 or 7 or 8 or 9 or 10 or 11 or 12 or 14 or 16 or 17 or 18
#20. (pump or delivery or release).mp.
#21. exp infusion pump/
#22. exp insulin infusion/
#23. 20 or 21 or 22
#24. glucose.mp.
#25. exp ambulatory monitoring/
#26. 24 and 25
#27. (monitor* or sensor* or sensing).mp.
#28. 24 and 27
#29. "sensed glucose".mp.
#30. (CGM or CGMS or glucosemeter or GlucoWatch or Guardian or Medtronic).mp.
#31. "freestyle navigator".mp.
#32. "glucose measurement".mp.
#33. exp blood glucose monitoring/
#34. 26 or 28 or 29 or 30 or 31 or 32 or 33

#35. (algorithm or computer or program* or modul* or controller or smartphone or tablet or "model predictive control" or MPC or "proportional-integral-derivative control" or "fuzzy logic" or FL).mp.

#36. 23 and 34 and 35

#37. 19 or 36

#38. crossover-procedure/ or double-blind procedure/ or randomized controlled trial/ or single-blind procedure/

#39. (random$ or factorial$ or crossover$ or cross over$ or cross-over$ or placebo$ or (doubl$ adj blind$) or (singl$ adj blind$) or assign$ or allocat$ or volunteer$).ti,ab,ot,hw.

#40. 38 or 39

#41. 37 and 40

#42. (letter or editorial or note).pt.

#43. animal/

#44. animal experiment/

#45. (rat or rats or mouse or mice or murine or rodent or rodents or hamster or hamsters or pig or pigs or porcine or rabbit or rabbits or animal or animals or dogs or dog or cats or cow or bovine or sheep or ovine or monkey or monkeys).ti,ab,ot,hw.

#46. or/43-45

#47. 42 or 46

#48. 41 not 47

Trial filter based on terms suggested by the Cochrane Handbook:

Lefebvre C, Manheimer E, Glanville J. Chapter 6: searching for studies. 6.3.2.2. What is in The Cochrane Central Register of Controlled Trials (CENTRAL) from EMBASE? In: Higgins JPT, Green S (editors). Cochrane Handbook for Systematic Reviews of Interventions Version 5.1.0 [updated March 2011]. The Cochrane Collaboration, 2011. Available from www.cochrane-handbook.org

COCHRANE

MeSH descriptor: [Pancreas, Artificial] explode all trees

MeSH descriptor: [Insulin Infusion Systems] explode all trees

MeSH descriptor: [Bionics] explode all trees

Exp blood glucose monitoring

MEDLINE (PubMed)

#1. Artificial pancreas [mh]

#2. Bioartificial Organs [mh] AND (pancreas [tw] OR insulin [tw] OR diabet* [tw])

#3. bionics [mh] AND (pancreas [tw] OR insulin [tw] OR diabet* [tw])

#4. “artificial pancreas” [tw]

#5. “bionic pancreas” [tw]

#6. “synthetic pancreas” [tw]

#7. “artificial endocrine pancreas” [tw]

#8. “artificial beta cell*” [tw]

#9. “artificial b cell*” [tw]

#10. “artificial b-cell*” [tw]

#11. closed-loop* [tw] AND (pancreas [tw] OR insulin [tw] OR diabet* [tw])

#12. “closed loop*” AND (pancreas [tw] OR insulin [tw] OR diabet* [tw])

#13. “bioartificial pancreas” [tw]

#14. “bio-artificial pancreas” [tw]

#15. OR/#1-14

#16. (pump [tw] OR delivery [tw] OR release [tw] OR Infusion Pumps, Implantable [mh] OR Insulin Infusion Systems [mh] OR Insulin/administration and dosage [mh])

#17. ((glucose [tw] AND Monitoring, Ambulatory [mh]) OR (glucose [tw] AND (monitor* [tw] OR sensor* [tw] OR sensing [tw])) OR "sensed glucose" [tw] OR CGM [tw] OR CGMS [tw] OR glucosemeter [tw] OR “freestyle navigator” [tw] OR GlucoWatch [tw] OR Guardian [tw] OR Medtronic [tw] OR Blood Glucose Self-Monitoring [mh] OR “glucose measurement” [tw])

#18. (algorithm [tw] OR computer [tw] OR program* [tw] OR modul* [tw] OR controller [tw] OR smartphone [tw] OR tablet [tw] OR “model predictive control” [tw] OR MPC [tw] OR “proportional-integral-derivative control” [tw] OR “fuzzy logic” [tw] OR FL [tw])

#19. AND/# 16-18

#20. #15 OR #19

#21. randomized controlled trial [pt]

#22. controlled clinical trial [pt]

#23. randomized [tiab]

#24. placebo [tiab]

#25. clinical trials as topic [mesh: noexp]

#26. randomly [tiab]

#27. trial [ti]

#28. #21 OR #22 OR #23 OR #24 OR #25 OR #26 OR #27

#29. animals [mh] NOT humans [mh]

#30. #28 NOT #29

#31. #20 AND #30

Trial filter based on terms suggested by the Cochrane Handbook:

Lefebvre C, Manheimer E, Glanville J. Chapter 6: searching for studies. 6.4.11 Box 6.4b. Cochrane Highly Sensitive Search Strategy for identifying randomized trials in MEDLINE: sensitivity- and precision-maximizing version (2008 revision); PubMed format. In: Higgins JPT, Green S (editors). Cochrane Handbook for Systematic Reviews of Interventions Version 5.1.0 [updated March 2011]. The Cochrane Collaboration, 2011. Available from www.cochrane-handbook.org

Appendix 4

Data extraction form

For every trial we extracted the following information:

Trial characteristics

Identifier

NCT

Source

Design

Setting

Population

Intervention characteristics

Pump

Sensor

Algorithm

Comparator

Duration

Baseline characteristics

Patients(n)

Age (SD)

Male (n)

Weight (SD)

BMI (SD)

Diabetes duration (SD)

Pump duration (SD)

HbA_{1c} (SD)

Daily insulin (SD)

We also extracted data (see below) for the following outcomes:

- % of overnight time glucose was between 3.9 – 10.0 mmol/l
- % of day and overnight time (24h) glucose was between 3.9 – 10.0 mmol/l
- % of overnight time glucose was below 3.9 mmol/l
- % of day and overnight time (24h) glucose was below 3.9 mmol/l
- % of overnight time glucose was above 10.0 mmol/l
- % of day and overnight time (24h) glucose was above 10.0 mmol/l
- Mean sensor blood glucose levels (24h)
- Mean sensor blood glucose levels (overnight)
- Change in HbA1c
- Insulin amount administered

CL arm pooled value

Mean

SD

Control arm pooled value

Mean

SD

Within pt diff (CL − Control intervention)

Mean

SD

Paired t test

p value

t value

We also extracted information for the following parameters for assessment of risk of bias for every individual trial:

- Sequence generation (or randomised treatment order for cross-over studies)
- Allocation concealment
- Blinding
- Dropout rate per arm/intervention period
- Type of analysis (ITT, per protocol) and method of imputation
- Selective outcome reporting
- Appropriateness of cross-over design
- Carry-over effects
- Unbiased data

Appendix 5

Overall risk of bias assessment

Key domains for assessment of risk of bias for the primary outcome

- Sequence generation (or randomised treatment order for cross-over studies)
- Allocation concealment
- Blinding
- Selective reporting
- Incomplete outcome data
- Other bias
  - Appropriateness of cross-over design (only for cross-over studies)
  - Carry-over effects (only for cross-over studies)
  - Unbiased data (only for cross-over studies)

The overall risk of bias was assessed in compliance with the following rules:

- If a study was considered at high risk of bias for any of the aforementioned domains, the study was characterised as "high risk study"
- If a study was considered at low risk of bias for all aforementioned domains, the study was characterised as "low risk study"
- In any other case the study was considered as "unclear risk study"

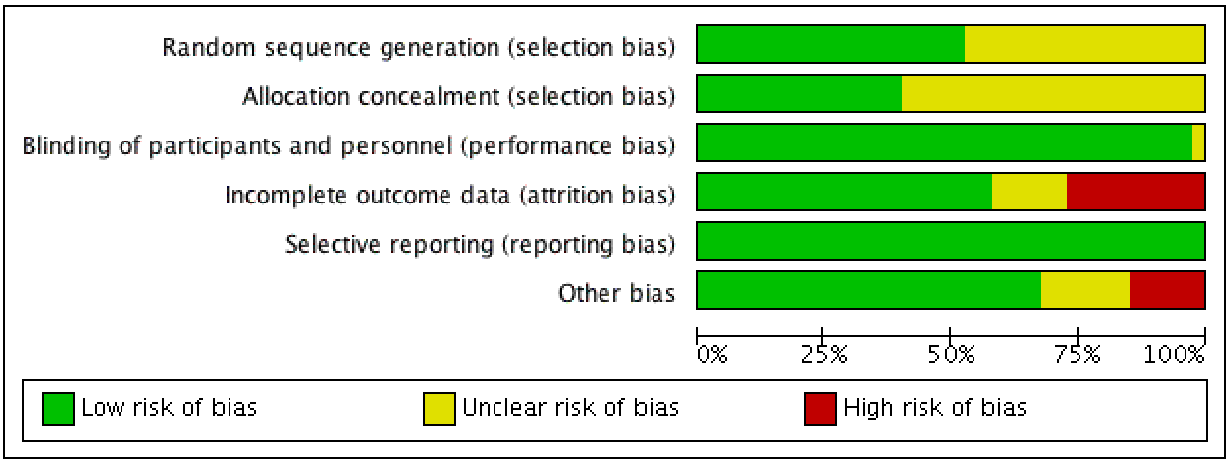

Appendix 6. Risk of bias graph: review authors' judgements about each risk of bias item presented as percentages across all included studies

	Random sequence generation (selection bias)	Allocation concealment (selection bias)	Blinding of participants and personnel (performance bias)	Incomplete outcome data (attrition bias)	Selective reporting (reporting bias)	Other bias
Bally 2017	+	+	+	+	+	+
Biester 2016	?	?	+	−	+	+
Blauw 2016	?	?	+	−	+	+
Breton 2017	?	?	+	+	+	+
Brown 2017	?	?	+	+	+	?
Chernavvsky 2016	?	?	+	+	+	?
De Bock 2015	?	?	+	+	+	?
DeBoer 2017	?	?	+	+	+	−
El-Khatib 2017	+	+	+	+	+	+
Favero 2016	?	?	+	+	+	+
Forlenza 2017a	?	?	+	+	+	+
Forlenza 2017b	+	+	+	+	+	−
Haidar 2015a	+	+	+	+	+	?
Haidar 2015b	+	+	+	+	+	?
Haidar 2016a	+	?	+	−	+	+
Haidar 2016b	+	?	+	−	+	+
Haidar 2017a	+	?	+	−	+	+
Haidar 2017b	+	?	+	−	+	+
Hovorka 2014	+	+	+	+	+	+
Kingman 2017	?	?	+	?	+	+
Kovatchev 2014	+	?	+	+	+	?
Kropff 2015	+	+	+	−	+	+
Leelarantha 2014	+	+	+	+	+	+
Ly 2014	?	?	+	−	+	−
Ly 2015a	?	?	+	?	+	+
Ly 2015b	?	?	?	?	+	+
Ly 2016a	?	?	+	?	+	+
Ly 2016b	?	?	+	−	+	−
Nimri 2014	+	+	+	−	+	+
Renard 2017	?	?	+	?	+	+
Russell 2014a	?	?	+	−	+	?
Russell 2014b	?	?	+	+	+	−
Russell 2016	+	+	+	+	+	+
Sharifi 2016	?	?	+	?	+	+
Spaic 2017	+	+	+	+	+	−
Tauschmann 2016a	+	+	+	+	+	+
Tauschmann 2016b	+	+	+	+	+	+
Thabit 2014	+	+	+	+	+	+
Thabit 2015a	+	+	+	+	+	+
Thabit 2015b	+	+	+	+	+	+

Appendix 7. Risk of bias summary: review authors' judgements about each risk of bias item for each included study.

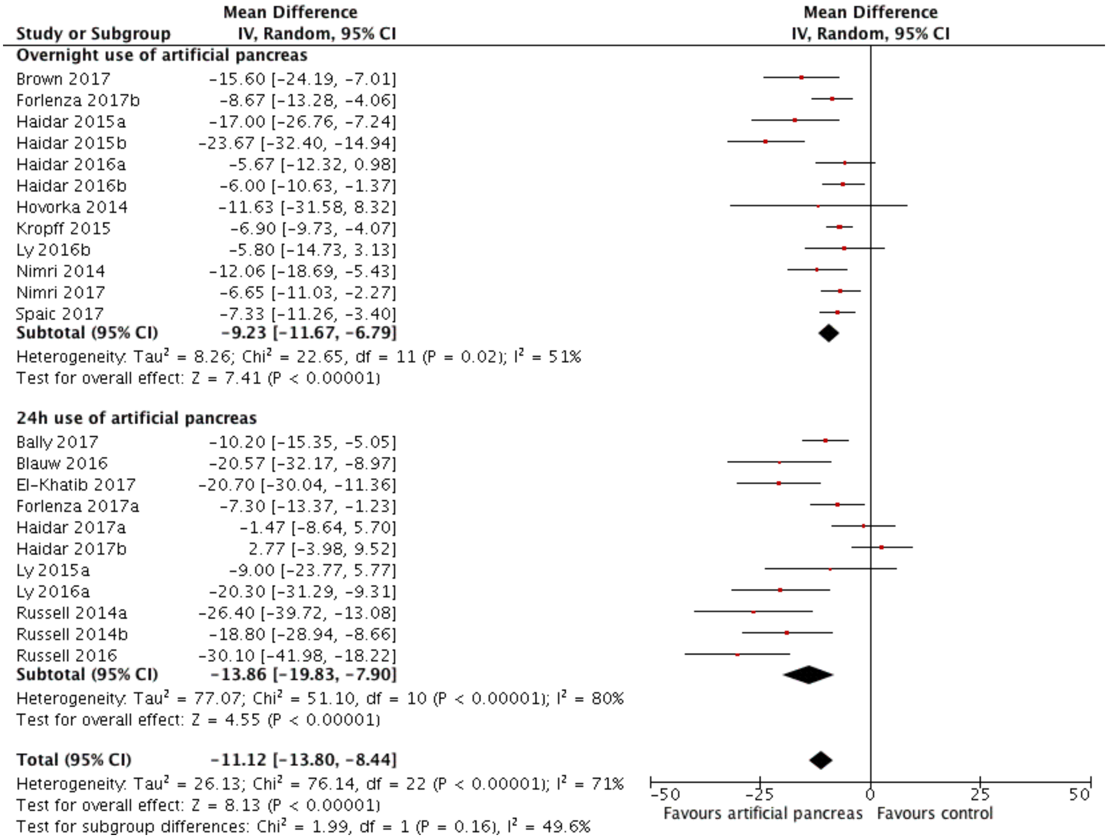

Appendix 8. Weighted mean difference in % of overnight time glucose was > 10.0 mmol/L. Artificial pancreas versus control treatment.

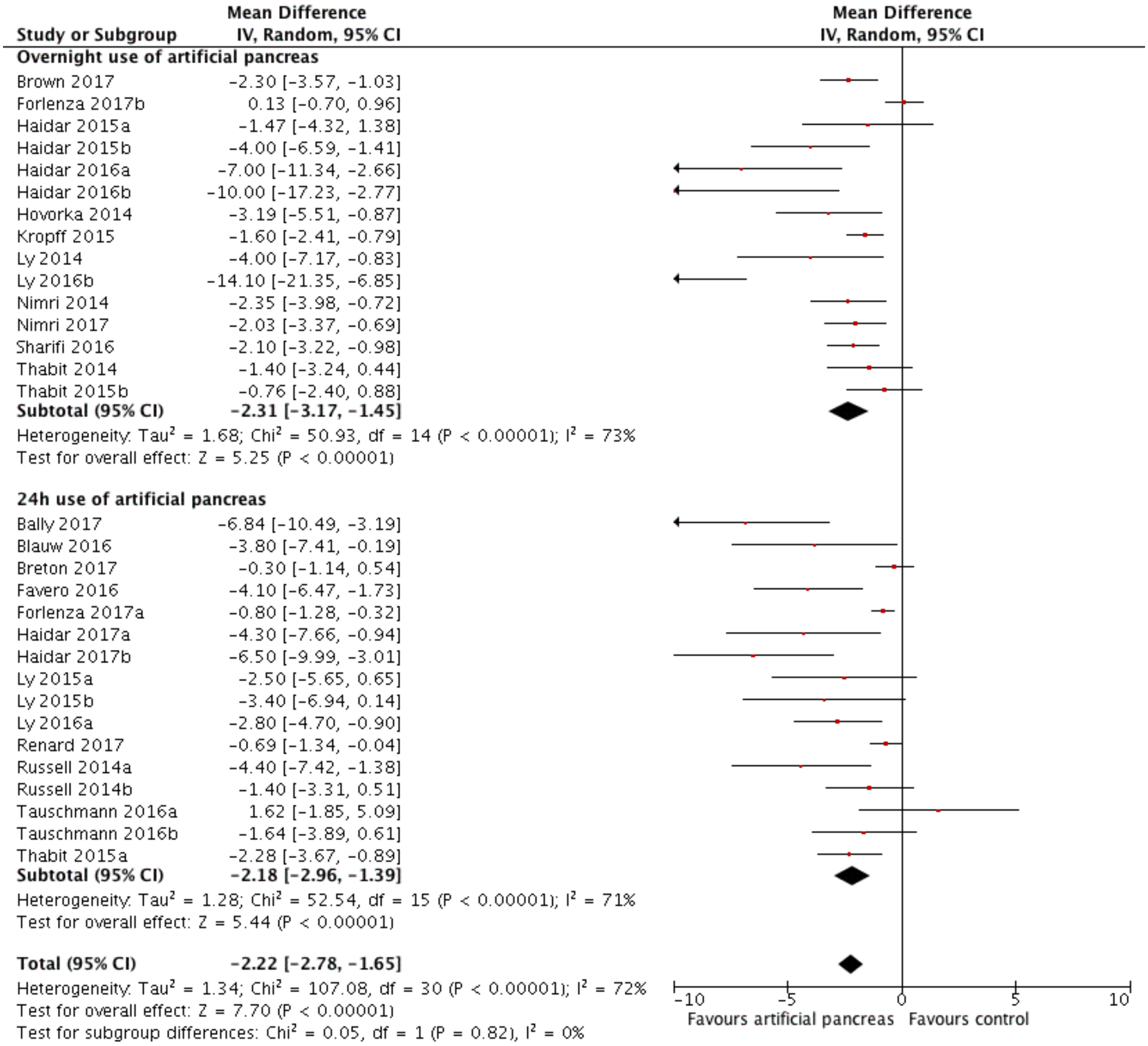

Appendix 9. Weighted mean difference in % of overnight time glucose was < 3.9 mmol/L. Artificial pancreas versus control treatment.

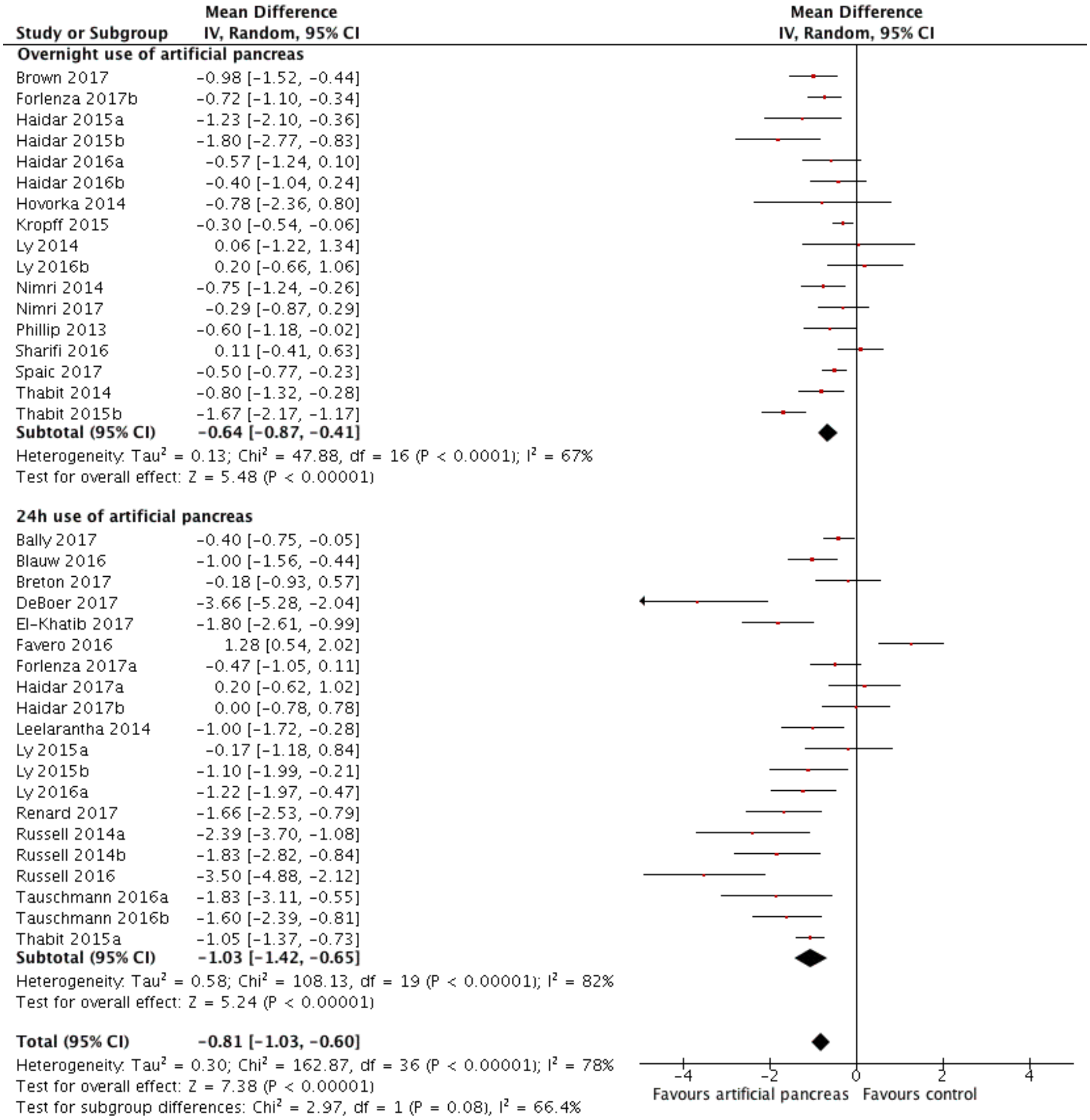

Appendix 10. Weighted mean difference in overnight mean sensor blood glucose (mmol/L). Artificial pancreas versus control treatment.

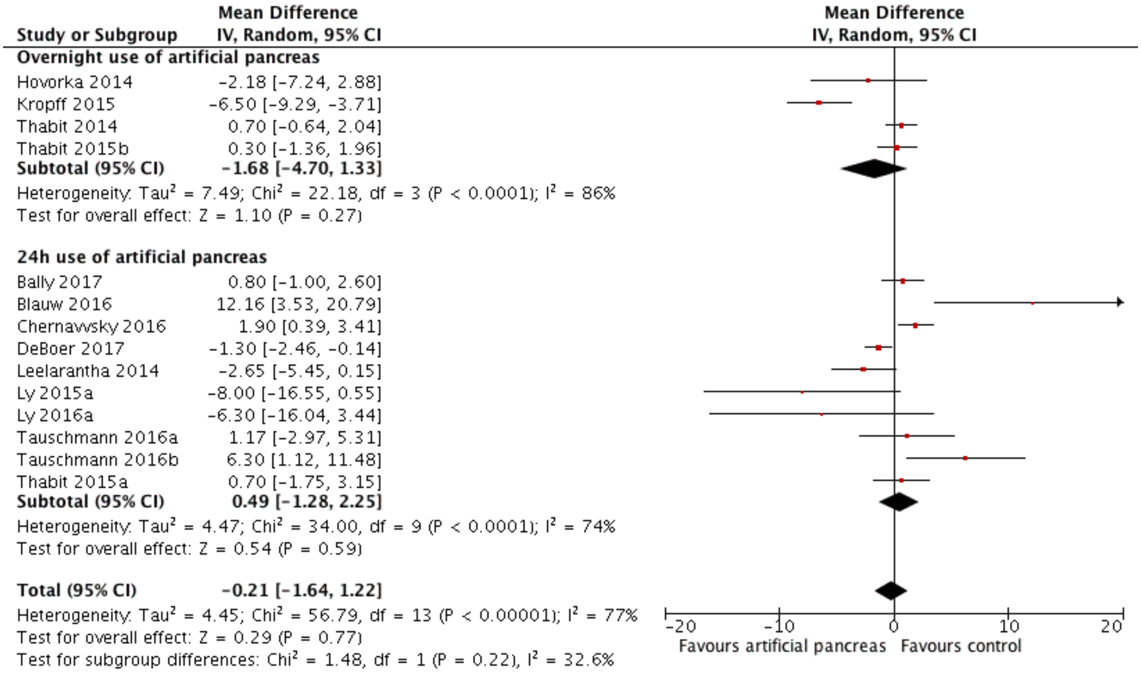

Appendix 11. Weighted mean difference in 24h insulin needs (IU). Artificial pancreas versus control treatment.

Appendix 12. Summary of findings of main analysis for all outcomes. Both overall effect estimates and subgroup effect estimates (based on overnight or 24h use of artificial pancreas system) between artificial pancreas and comparator are presented. BG: blood glucose. CIs: confidence intervals. AP: Artificial pancreas. LBGI: low blood glucose index. NE: not estimable.

Outcome	Number of studies	Effect estimate	Der Simmonian Laird 95% CIs	95% Hartung-Knapp CIs	95% Prediction intervals	I^2 (%)	Tau^2
% of 24h time between 3.9 – 10.0 mmol/L, Overall effect estimate	32	9.62	7.54 to 11.7	7.83 to 12.41	-0.63 to 19.87	78	24.09
% of 24h time between 3.9 – 10.0 mmol/L, Overnight use of AP	7	7.16	5.73 to 8.58	5.16 to 9.16	5.29 to 9.02	0	0
% of 24h time between 3.9 – 10.0 mmol/L, 24h use of AP	25	10.79	7.88 to 13.7	8.47 to 13.11	-2.52 to 24.1	81	39.21
% of overnight time between 3.9 – 10.0 mmol/L, Overall effect estimate	31	15.15	12.21 to 18.09	12.57 to 17.73	1.31 to 28.98	73	43.48
% of overnight time between 3.9 – 10.0 mmol/L, Overnight use of AP	14	14.25	11.13 to 17.37	10.46 to 18.04	4.04 to 24.45	63	19.39
% of overnight time between 3.9 – 10.0 mmol/L, 24h use of AP	17	16.44	10.88 to 22.01	12.51 to 20.37	-5.68 to 38.56	78	99.63
% of 24h time above 10.0 mmol/L, Overall effect estimate	22	-8.52	-11.14 to -5.9	-11.83 to -5.21	-20.09 to 3.05	80	28.98
% of 24h time above 10.0 mmol/L, Overnight use of AP	3	-6	-8.4 to -3.6	-7.94 to -4.06	-21.55 to 9.55	0	0
% of 24h time above 10.0 mmol/L, 24h use of AP	19	-9.08	-12.23 to -5.93	-12.98 to -5.18	-22.44 to 4.28	83	37.53
% of overnight time above 10.0 mmol/L, Overall effect estimate	23	-11.12	-13.8 to -8.44	-13.92 to -8.32	-22.12 to -0.11	71	26.13
% of overnight time above 10.0 mmol/L, Overnight use of AP	12	-9.23	-11.67 to -6.79	-12.68 to -5.78	-16.2 to -2.25	51	8.26

% of overnight time above 10.0 mmol/L, 24h use of AP	11	-13.86	-19.83 to -7.9	-19.69 to -8.03	-34.87 to 7.15	80	77.07
% of 24h time below 3.9 mmol/L, Overall effect estimate	29	-1.49	-1.86 to -1.11	-1.91 to -1.07	-3.11to 0.13	74	0.59
% of 24h time below 3.9 mmol/L, Overnight use of AP	7	-1.1	-1.46 to -0.75	-1.58 to -0.62	-1.55 to -0.64	0	0
% of 24h time below 3.9 mmol/L, 24h use of AP	22	-1.64	-2.12 to -1.16	-2.21 to -1.07	-3.56 to 0.28	80	0.79
% of overnight time below 3.9 mmol/L, Overall effect estimate	29	-2.22	-2.78 to -1.65	-2.86 to -1.58	-4.66 to 0.22	72	1.34
% of overnight time below 3.9 mmol/L, Overnight use of AP	15	-2.31	-3.17 to -1.45	-3.5 to -1.12	-5.2 to 0.64	73	1.68
% of overnight time below 3.9 mmol/L, 24h use of AP	14	-2.18	-2.96 to -1.39	-2.89 to -1.47	-4.79 to 0.43	71	1.28
Overnight LBGI, Overall effect estimate	11	-0.37	-0.56 to -0.18	-0.61 to -0.13	-0.97 to 0.22	85	0.06
Overnight LBGI, Overnight use of AP	9	-0.29	-0.47 to -0.11	-0.52 to -0.06	-0.86 to 0.28	84	0.05
Overnight LBGI, 24h use of AP	2	-1.05	-1.53 to -0.57	-1.54 to - 0.56	NE	0	0
24h Mean BG (mmol/L), Overall effect estimate	32	-0.48	-0.66 to -0.3	-0.7 to -0.26	-1.36 to 0.4	84	0.18
24h Mean BG (mmol/L), Overnight use of AP	6	-0.29	-0.43 to -0.16	-0.48 to -0.1	-0.62 to 0.04	20	0.01
24h Mean BG (mmol/L), 24h use of AP	26	-0.54	-0.78 to -0.31	-0.82 to -0.26	-1.65 to 0.57	87	0.28
Overnight Mean BG (mmol/L), Overall effect estimate	35	-0.81	-1.03 to -0.6	-0.95 to -0.67	-1.94 to 0.3	78	0.3

Overnight Mean BG (mmol/L), Overnight use of AP	17	-0.64	-0.87 to -0.41	-0.91 to -0.37	-1.44 to 0.16	67	0.13
Overnight Mean BG (mmol/L), 24h use of AP	18	-1.03	-1.42 to -0.65	-1.53 to -0.53	-2.69 to 0.63	82	0.58
24h Total insulin delivered (IU), Overall effect estimate	14	-0.21	-1.64 to 1.22	-2.32 to 1.9	-5.07 to 4.65	77	4.45
24h Total insulin delivered (IU), Overnight use of AP	4	-1.68	-4.7 to 1.33	-7.07 to 3.71	-15.18 to 11.82	86	7.49
24h Total insulin delivered (IU), 24h use of AP	10	0.49	-1.28 to 2.25	-2.22 to 3.2	-4.8 to 5.78	74	4.47
HbA1c	3	-0.26	-0.38 to -0.13	-0.41 to -0.11	-1.10 to 0.58	0	0

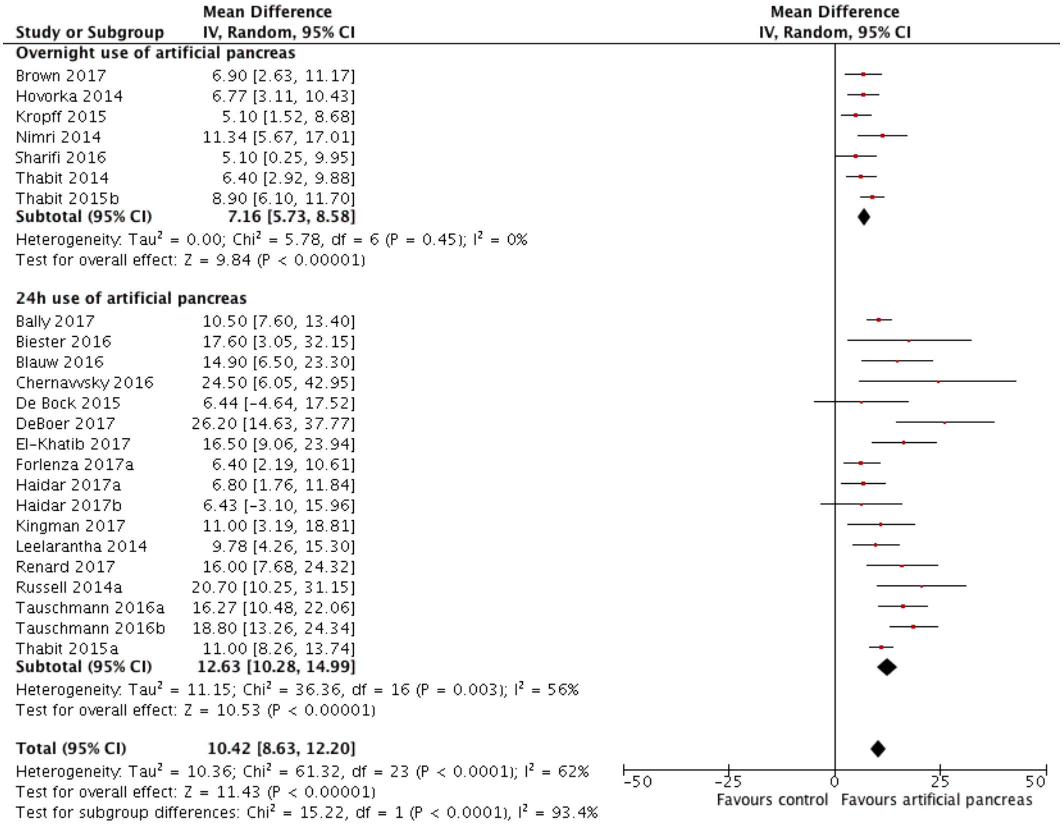

Appendix 13. Weighted mean difference in % of 24h time in near normoglycaemic range (3.9 – 10.0 mmol/L). Artificial pancreas versus control treatment. Sensitivity analysis excluding trials recruiting patients in camps.

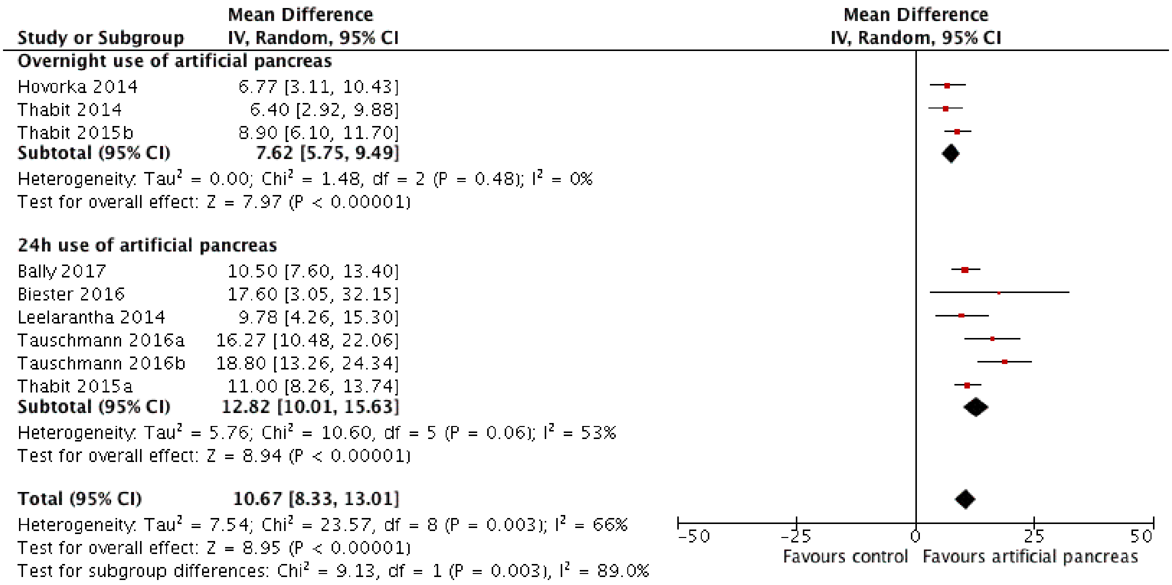

Appendix 14. Weighted mean difference in % of 24h time in near normoglycaemic range (3.9 – 10.0 mmol/L). Artificial pancreas versus control treatment. Sensitivity analysis including only trials recruiting unsupervised patients in free-living conditions.

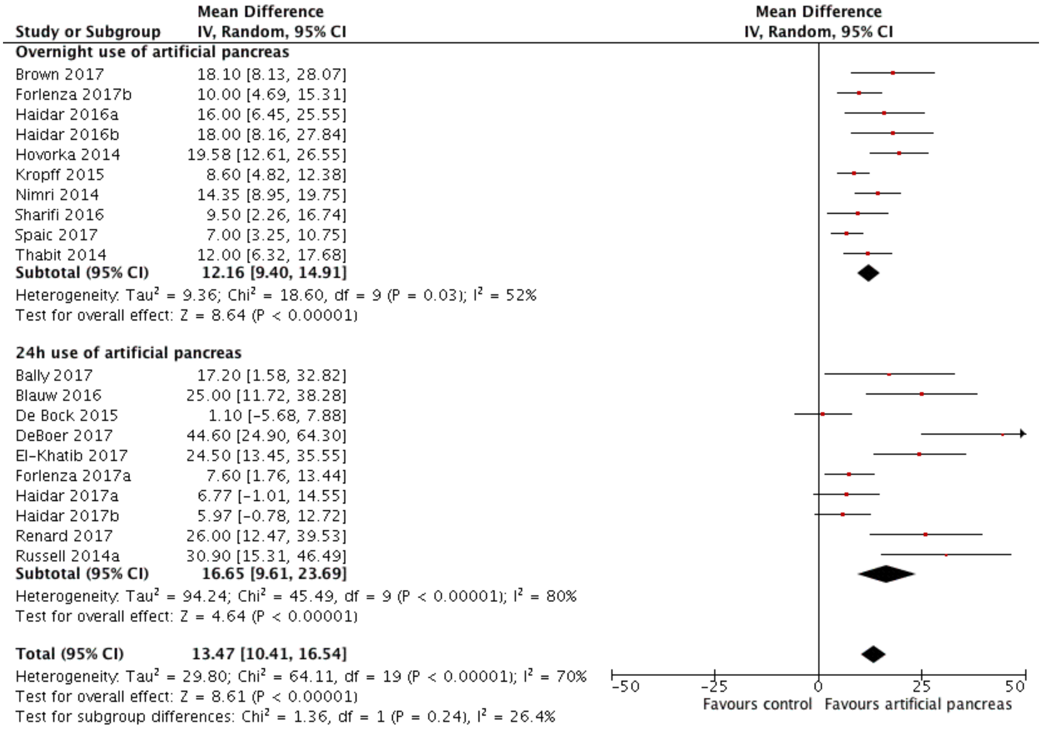

Appendix 15. Weighted mean difference in % of overnight time in near normoglycaemic range (3.9 – 10.0 mmol/L). Artificial pancreas versus control treatment. Sensitivity analysis excluding trials recruiting patients in camps.

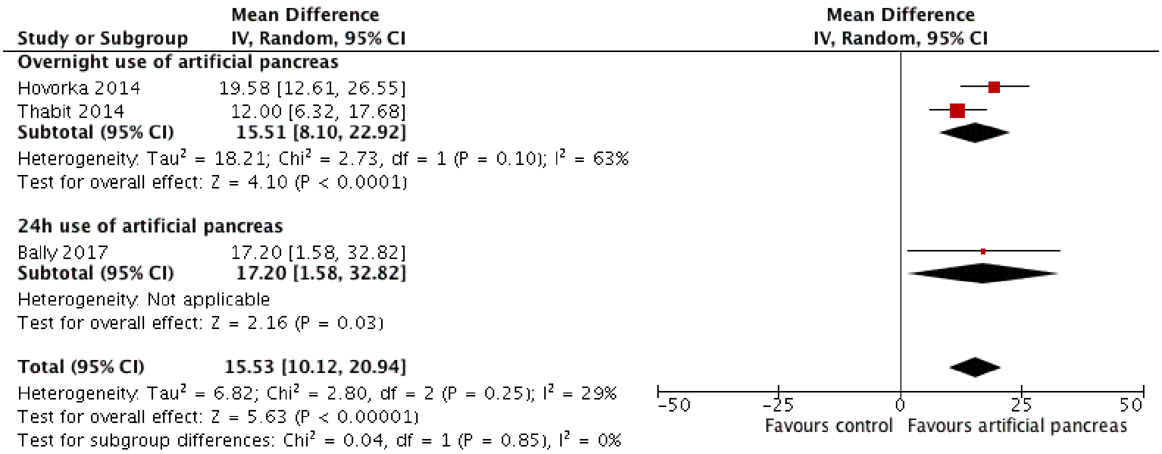

Appendix 16. Weighted mean difference in % of overnight time in near normoglycaemic range (3.9 – 10.0 mmol/L). Artificial pancreas versus control treatment. Sensitivity analysis including only trials recruiting unsupervised patients in free-living conditions.

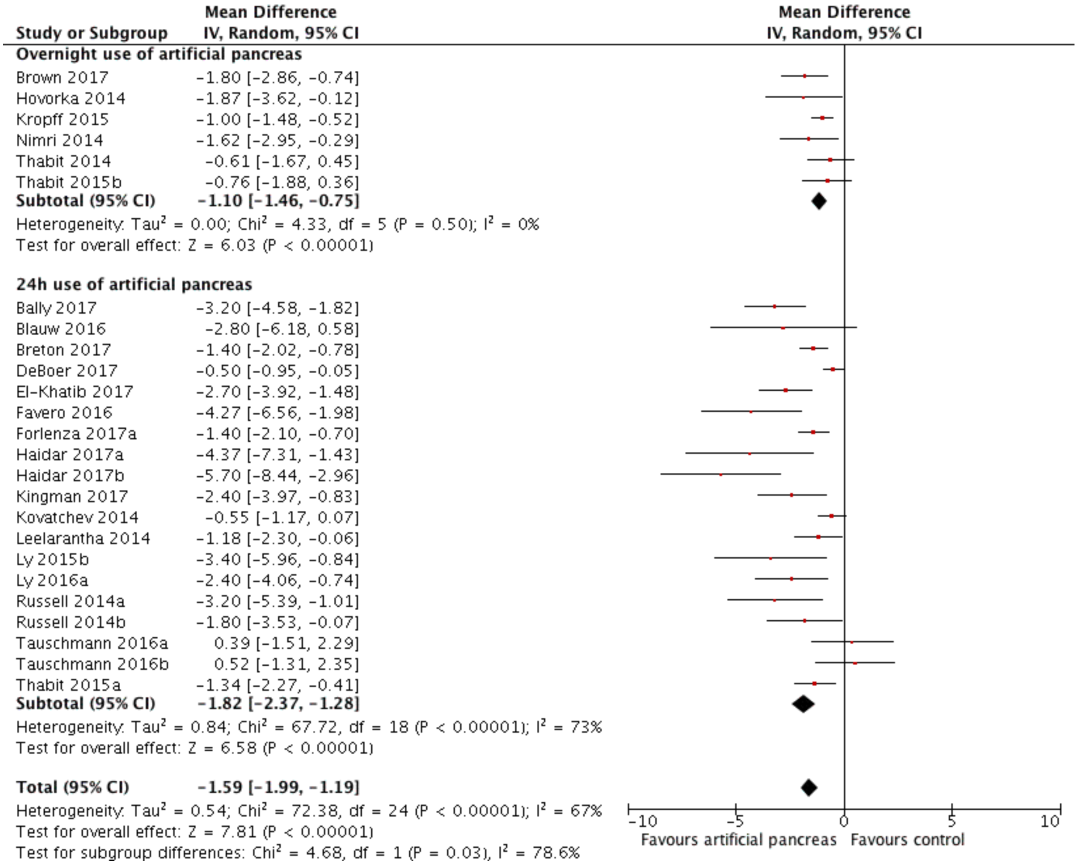

Appendix 17. Weighted mean difference in % of 24h time glucose was < 3.9 mmol/L. Artificial pancreas versus control treatment. Sensitivity analysis excluding trials comparing artificial pancreas systems with low glucose suspend (LGS) systems.

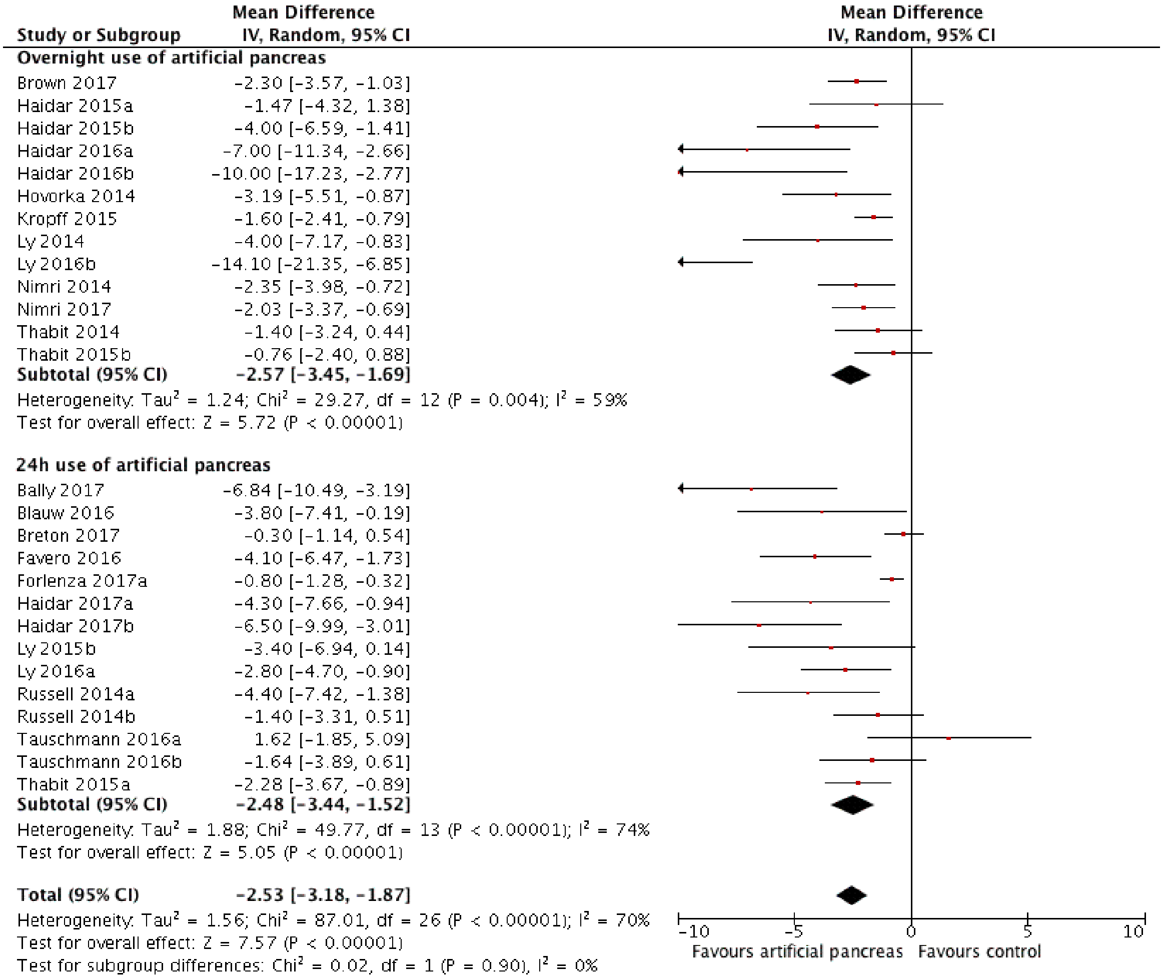

Appendix 18. Weighted mean difference in % of overnight time glucose was < 3.9 mmol/L. Artificial pancreas versus control treatment. Sensitivity analysis excluding trials comparing artificial pancreas systems with low glucose suspend (LGS) systems.

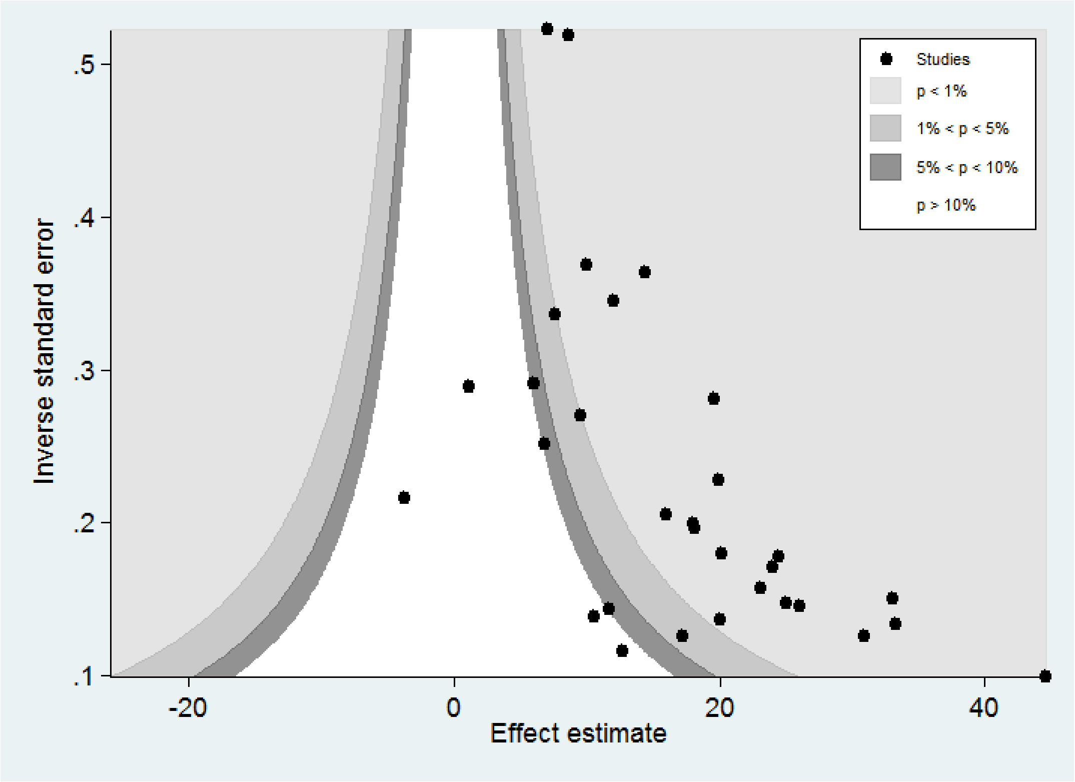

Appendix 19. Contour-enhanced funnel plot for studies assessing overnight time spent in near normoglycaemia.
